# Spasticity treatment patterns among people with multiple sclerosis: a Swedish cohort study

**DOI:** 10.1136/jnnp-2022-329886

**Published:** 2022-12-20

**Authors:** Kelsi A Smith, Fredrik Piehl, Tomas Olsson, Lars Alfredsson, Jan Hillert, Ingrid Kockum, Pernilla Stridh, Scott Montgomery

**Affiliations:** 1 Clinical Epidemiology Division, Department of Medicine Solna, Karolinska Institute, Stockholm, Sweden; 2 Clinical Neuroscience, Karolinska Institute, Stockholm, Sweden; 3 Centre of Molecular Medicine, Karolinska University Hospital, Stockholm, Sweden; 4 Academic Specialist Centre, Centre of Neurology, SLSO, Stockholm, Sweden; 5 Centre of Occupational and Environmental Medicine, Region Stockholm, Stockholm, Sweden; 6 Centre for Molecular Medicine, Karolinska University Hospital, Stockholm, Sweden; 7 Clinical Epidemiology and Biostatistics, School of Medical Sciences, Faculty of Medicine and Health, Örebro University, Orebro, Sweden; 8 Department of Epidemiology and Public Health, University College London, London, UK

**Keywords:** spasticity, multiple sclerosis, clinical neurology, epidemiology

## Abstract

**Background:**

Spasticity is common among people with multiple sclerosis (MS), but there are few studies of spasticity treatment patterns. We aim to describe associations with spasticity treatment measured primarily by oral baclofen use.

**Methods:**

This cohort study using Swedish registers included 1826 and 3519 people with incident and prevalent MS (pwIMS, pwPMS) respectively, followed from 2005 to 2014. Cox regression assessed factors associated with new baclofen prescriptions and its discontinuation.

**Results:**

A total of 10% of pwIMS and 19% of pwPMS received baclofen, a drug prescribed specifically for spasticity in Sweden, of which many patients had relapsing-remitting course. Prescriptions occurred soon after MS diagnosis: pwIMS received baclofen typically within 6 months of diagnosis, and pwPMS within 3 years. Younger patients compared with older patients were three times more likely to receive baclofen with similar disability level measured using Expanded Disability Severity Scores (EDSS). Patients aged 18–44 years with EDSS 3.0–5.0 have an HR for baclofen use of 5.62 (95% CI 2.91 to 10.85) and EDSS 6+ have an HR of 15.41 (95% CI 7.07 to 33.58) compared with individuals with EDSS 0–2.5. In comparison, patients aged 45+ years with EDSS 3.0–5.0 have an HR of 2.05 (95% CI 1.10 to 3.82) and EDSS 6+ an HR of 4.26 (95% CI 1.96 to 9.17). Baclofen discontinuation was high: 49% (95% CI 0.42 to 0.57) of pwIMS discontinued within 150 days of dispensation, 90% discontinued within 2 years including patients with progressive course or higher EDSS. Associations among pwPMS and sensitivity analyses including additional treatments were similar.

**Conclusions:**

Younger patients with MS are more likely to receive baclofen compared with older patients with MS. High rates of baclofen discontinuation highlight the need for more tolerable and efficacious spasticity treatments and monitoring of spasticity among people with MS.

**WHAT IS ALREADY KNOWN ON THIS TOPIC**

- Studies of spasticity among people with multiple sclerosis (MS) are few. Factors increasing the need for spasticity treatment are largely unknown given the impact of spasticity in the day-to-day lives of people with MS.

**WHAT THIS STUDY ADDS**

- This is the first large, population-based assessment of spasticity treatment following patients over 10 years and shows that younger, newly diagnosed people with MS are prescribed baclofen to treat spasticity, especially those with greater disability compared with older patients with MS. This is the first study to show high rates of baclofen discontinuation among all patients with MS as more than half stopped treatment within 6 months.

**HOW THIS STUDY MIGHT AFFECT RESEARCH, PRACTICE OR POLICY**

- Spasticity may occur earlier among patients with MS than previously thought, which is of importance for treating clinicians managing MS symptoms. High rates of baclofen discontinuation demonstrates a low success rate of treating spasticity and demonstrates an unmet need of tolerated, effective spasticity treatments. This study provides evidence for the timing of treatment, and highlight issues with long-term treatment using baclofen to manage spasticity.

## Introduction

Spasticity is a result of changes in muscular stretch reflexes^1^ and is a complication of multiple sclerosis (MS) possibly reflecting disease severity and progression, or individual susceptibility. Cross-sectional, register-based studies of individuals with prevalent MS in Germany and North America report spasticity prevalence between 53%^2^ and 60%^3^ to greater than 80%^4^ of varying severity, with increases in spasticity severity associated with a progressive MS disease course.^3 4^ Spasticity can present with a range of symptoms even among individuals with a similar level of functioning.^4 5^ Variation in levels of severity can occur even over a short period of time and spasticity is present commonly in the lower extremities among people with MS (pwMS).^4 6 7^ Spasticity has an impact on many aspects of an individual’s life including quality of life, mobility,^3^ pain^8^ and leads to increased disability.^4^

The specific pathophysiology of spasticity among pwMS is unknown. One hypothesis postulates spasticity is due to lesions in upper motor neuron pathways in the central nervous system. This causes increases in muscular reflex stretches and muscle tone similar to spasticity observed among diseases involving upper motor neuron syndromes.^1 7^ Treatments for spasticity vary by country, often involving a multidisciplinary approach.^9^ Oral baclofen is the only approved first-line pharmacological treatment for spasticity in Sweden, while the cannabinoid Sativex is approved for moderate to severe spasticity with insufficient response to other lines of therapy. As Sativex is not reimbursed in Sweden, its use is very limited. Baclofen can be delivered in tablet form or intrathecally, although intrathecal baclofen pumps are reserved as a second-line treatment for severe spasticity. Certain other oral drug therapies are used off-label to treat spasticity, including diazepam, clonazepam and gabapentin.^10^ More rarely used drug therapies include tizanidine, dantrolene or botulinum toxin A injections.^10^

Studies of spasticity among pwMS are rare and mostly involved patients with prevalent MS using a cross-sectional study design, assess spasticity using self-reported data or were clinical trials. Studies using self-assessed spasticity among patients with prevalent MS found that most often spasticity affects males, individuals with progressive forms of MS, and older individuals.^4 6^ Conditions such as seizures and stroke are associated with spasticity among people with prevalent MS.^4 6^ Disease-modifying therapies (DMTs) and their effects on spasticity are not well studied, although beta-interferon may exacerbate spasticity.^7^ Clinical guidelines are often based on clinical-trial data, and there is no comprehensive study of treatment patterns using objective measures among a wide-range of patients with MS in the real-world context. Understanding how and why spasticity manifests frequently among pwMS will help identify possible preventative strategies and earlier treatment possibilities. Our aim was to determine associations with a proxy measure of spasticity, use of oral baclofen among people with incident and prevalent MS (pwIMS, pwPMS) diagnoses using Swedish national registers.

## Methods

### Study setting and population

The follow-up for this cohort study was from 1 July 2005 to 31 December 2014 and linked data from Swedish registers to epidemiological case–control studies of MS in Sweden: the Epidemiological Investigation of MS (EIMS), Genes and Environment in MS (GEMS), and Immunomodulation and MS Study (IMSE) described elsewhere.^11^

**Figure 1:**
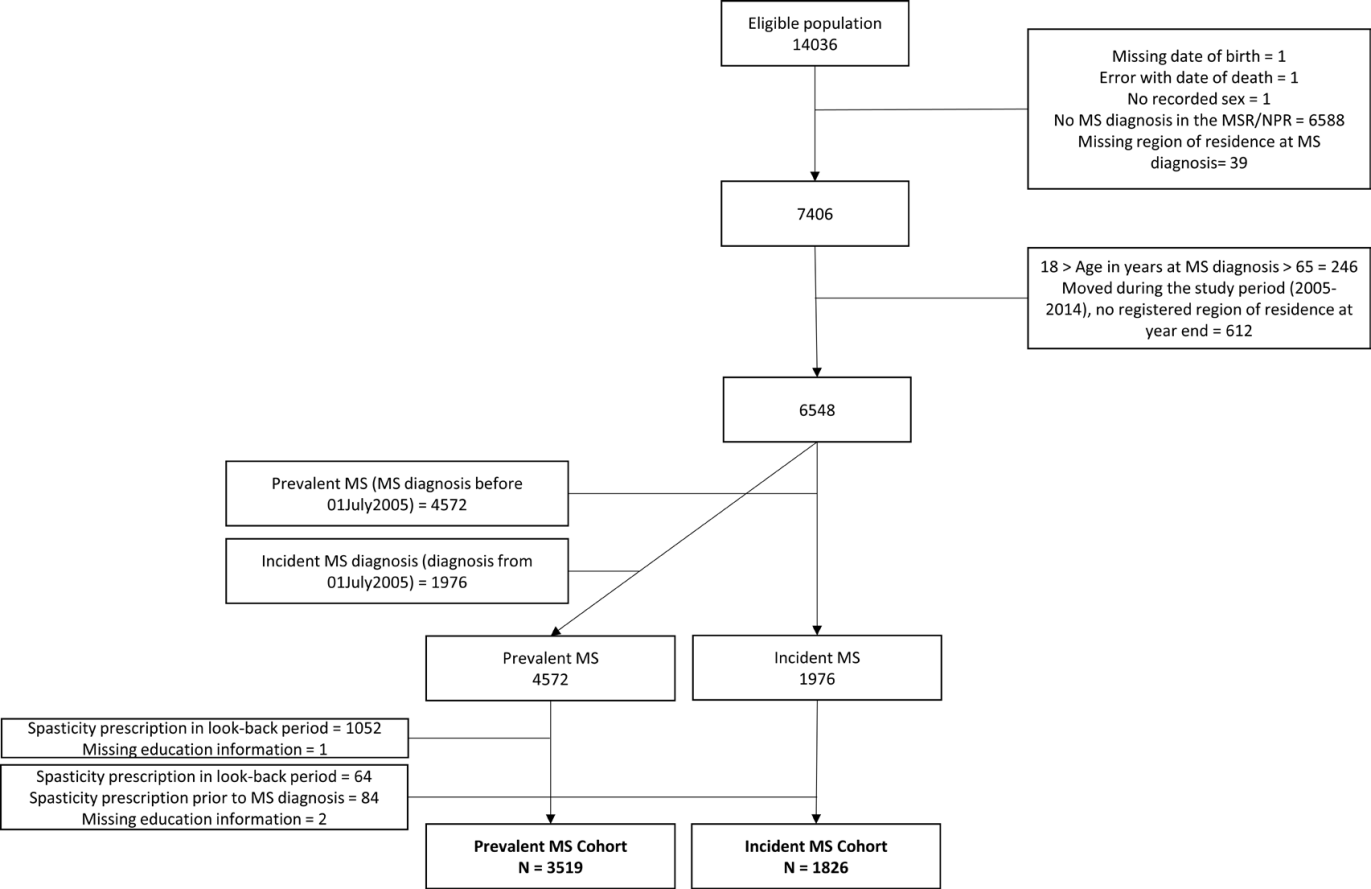
Participant flow chart for the prevalent and incident multiple sclerosis (MS) cohorts. Errors with date of death are due to reassignment of personal numbers to another person after death of an individual. MSR, Multiple Sclerosis Register, NPR, National Patient Register.

The study population comprised pwMS consenting to participation in EIMS, GEMS or IMSE between 2004 and 2014 that were genotyped. A person was identified as having MS if they had any record in the Swedish Multiple Sclerosis Register (SMSReg) (a register containing information for 85% of the MS population recording MS-specific clinical variables since 1996^12^), or if they had two or more International Classification of Disease (ICD)-10 codes for MS (G35.0, G35.9) at least 180 days apart in the National Patient Register (NPR). This NPR definition has good positive predictive value when compared with the SMSReg^13–15^ and the SMSReg has a high level of diagnostic specificity.^12^ Previously identified controls fulfilling MS criteria were included as pwMS. Where an MS diagnosis was recorded in both the SMSReg and NPR, the earliest diagnosis date was used. If diagnosis date was missing, onset date from the SMSReg was used. Individuals diagnosed with MS between 18 and 65 years of age were included. Additional inclusion/exclusion criteria are described in figure 1.

**Figure 2:**
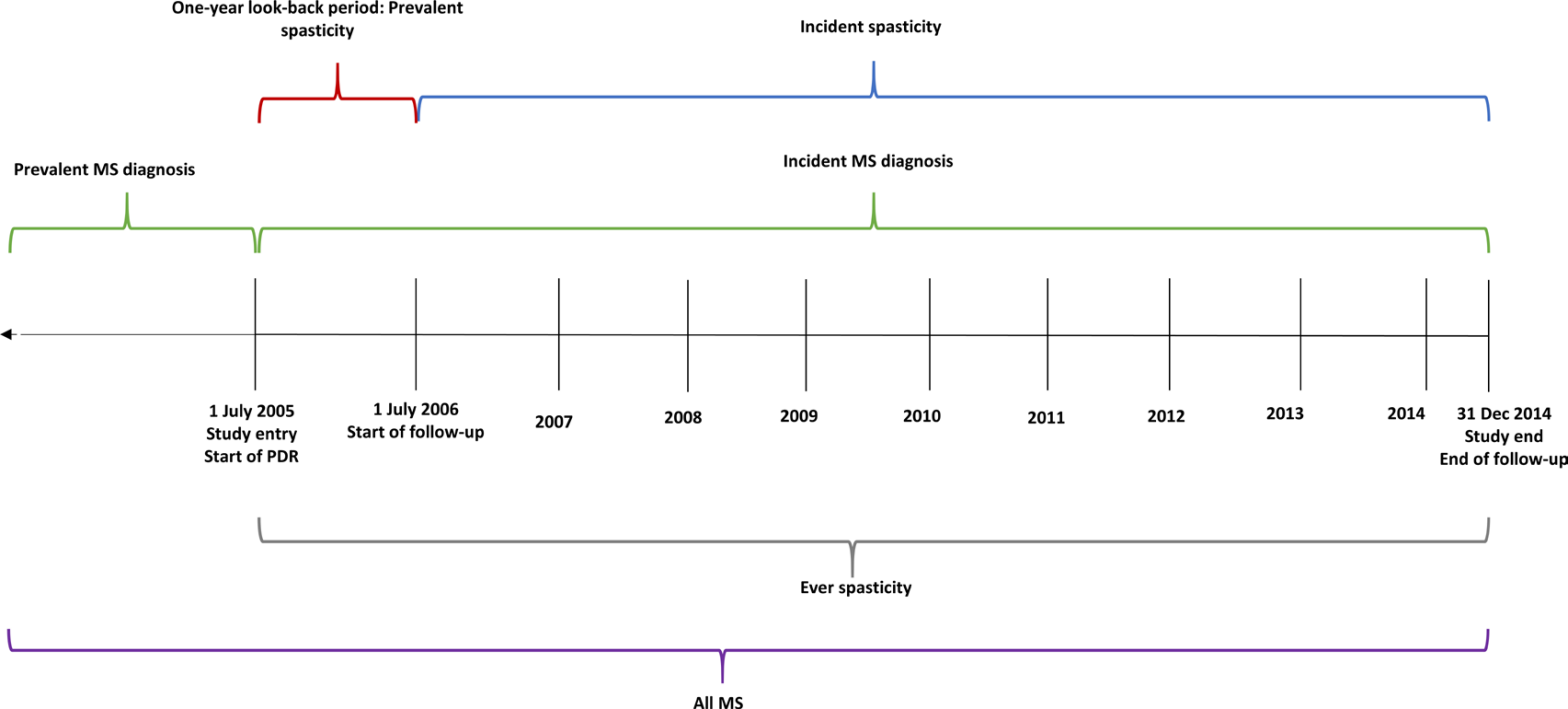
Graphical depiction of how cohort populations and spasticity treatment definitions were defined. Study entry and study end is the same among both prevalent and incident multiple sclerosis (MS) diagnoses. The start of follow-up from 1 July 2006 is only applicable to people with prevalent and incident multiple sclerosis who do not have a spasticity treatment recorded in the 1-year look-back period (1 July 2005–30 June 2006). Start of follow-up for people with incident multiple sclerosis began from their multiple sclerosis diagnosis date. PDR, Prescribed Drug Register.

Two cohorts were defined: pwPMS and pwIMS. People diagnosed prior to 1 July 2005 were considered as pwPMS, and after 1 July 2005 were considered as pwIMS (figure 2).

### Outcome

Dispensed medications from all pharmacies in Sweden are recorded in the Prescribed Drug Register (PDR) which started from 1 July 2005.^16^ Date of first baclofen dispensation in the PDR from 1 July 2005 to 31 December 2014 was identified using Anatomical Therapeutic Chemical (ATC) code M03BX01. Individuals with possible spasticity treatments before MS diagnosis were excluded [ATC codes: baclofen (M03BX01), diazepam (N05BA1), clonazepam (N03AE01), gabapentin (N03AX12) or cannaboids (N02BG10)]. A 1-year look-back period from 1 July 2006 to 1 July 2005 was used to exclude individuals with prevalent spasticity treatments as defined above (figure 2) to account for delay of prescriptions being recorded nationally, or to allow individuals with a prior prescription to refill.

All possible spasticity treatments (all drugs named above) within the same time-period and using the same exclusion criteria as defined above, although less specific for spasticity, were also considered. This secondary outcome was defined as the date of first of any possible spasticity treatment.

### MS variables

Individuals were categorised as relapsing-remitting MS (RRMS), secondary progressive MS (SPMS) and primary progressive MS (PPMS, including primary relapsing) at the start of follow-up (1 July 2006). Individuals with a missing year of diagnosis change were considered to have SPMS from study entry and date of diagnosis change was the midpoint of the year when they were registered as SPMS (15 June).

DMTs considered as highly effective were: alemtuzumab, ciclosporin, cladribine, daclizumab, dimethyl-fumarate, fingolimod, natalizumab, ofatumumab, rituximab and teriflunomide (although not all were used in this cohort). Moderately effective DMTs were interferons and glatiramer acetate. Date of first DMT use was the earliest recorded treatment date.

Relapse dates were extracted and records with missing/erroneous dates (ie, dates before birth date), or dates 3 years prior to MS onset were excluded. Relapses within 14 days of one another were considered the same episode.

Expanded Disability Severity Scale (EDSS) scores and date of changes were categorised as 0–2.5, 3.0–5.5 and 6+ due to the large number of score changes. Baseline EDSS was defined as the score closest in time to start of follow-up, provided it was not greater than 90 days after start of follow-up.

### Potential risk factors

Diseases associated with spasticity among pwPMS were identified prior to baclofen initiation using the NPR (1 January 1997 to study-end) using ICD-10 codes and/or the PDR (from 1 July 2005 to study-end) using ATC codes further described in online supplemental table 0.

10.1136/jnnp-2022-329886.supp1 Supplementary data

### Other variables

Highest-ever attained education was determined from LISA^17^ and categorised as compulsory, postcompulsory and tertiary. Using the Total Population Register,^18^ birth date was estimated as the midpoint of the month of birth (15th day), and region of residence at MS diagnosis was determined.

### Statistical analysis

#### Time to event

Follow-up for pwIMS started from MS diagnosis date and pwPMS from 1July 2006. End of follow-up was 31 December 2014, death (identified from the Cause of Death Register^19^), or date of first baclofen initiation (primary outcome), whichever came first. Cox regression models used age as the underlying timescale and provided HRs for baclofen initiation. Models were adjusted for region of residence at MS diagnosis, education and other variables. Time from MS diagnosis was used as a secondary timescale. Disease course, DMTs and EDSS were used as time-varying variables and analyses including these variables were restricted to individuals with complete information. Proportional hazards were assessed using Schoenfeld residuals or with an interaction by timescale. Variables with non-proportional hazards were included using either Stratified Cox regression models, or with an interaction with the underlying timescale.

#### Sensitivity analyses

Further Cox regression models were used to examine associations with: first treatment among all possible spasticity treatments (secondary outcome); secondary outcome excluding individuals with depression and secondary outcome excluding individuals with seizures. These exclusions were conducted as the primary indication of spasticity treatments other than baclofen are seizure/epilepsy or neuropathic pain.

#### Discontinuation

Time to discontinuation of spasticity treatment was assessed using failure functions for primary and secondary outcomes. For secondary outcomes, individual drugs were stratified due to differences in prescription patterns. Follow-up began from the first day of treatment dispensation until discontinuation of treatment, death or study end. Discontinuation was defined as no additional dispensation within the time from first dispensation plus gap days of possible treatment durations (90, 150 or 180 days) to account for prescribing variation for different drugs.

Data management was performed using SAS V.9.4^20^ and analysis using STATA V.16.^21^

## Results

Some 1826 and 3519 pwIMS and pwPMS were included and were mainly females (table 1). pwPMS were generally older, had more severe MS, had more comorbid diseases and used a greater proportion of moderately effective DMTs than pwIMS.

**Table 1:** Baseline characteristics assessed at study entry among incident and prevalent MS individuals, stratified by baclofen treatment status

Baseline characteristics	Incident MS N=1826		Prevalent MS N=3519	
	No Baclofen	Baclofen	No Baclofen	Baclofen
N (%)	1638	188 (10.29)	2891	628 (17.84)
Sex (%)				
Male	437 (26.68)	72 (38.30)	695 (24.04)	207 (32.96)
Female	1201 (73.32)	116 (61.70)	2196 (75.96)	421 (67.04)
Highest attained education (%)				
Primary education	132 (8.06)	21 (11.17)	364 (12.59)	98 (15.61)
Secondary education	767 (46.83)	97 (51.60)	1341 (46.39)	280 (44.59)
Tertiary education	739 (45.12)	70 (37.23)	1186 (41.02)	250 (39.81)
Comorbid diseases (%)*				
Depression	290 (17.70)	44 (23.40)	682 (23.59)	198 (31.53)
Concussion or TBI	3 (0.18)	0 (0.00)	8 (0.28)	0 (0.00)
Coronary artery disease	2 (0.12)	0 (0.00)	22 (0.76)	6 (0.96)
Vascular disease	19 (1.16)	3 (1.60)	88 (3.04)	24 (3.82)
Stroke	12 (0.73)	4 (2.13)	50 (1.73)	9 (1.43)
Parkinson’s disease	5 (0.31)	0 (0.00)	9 (0.31)	6 (0.96)
Diabetes				
No diabetes	1625 (99.21)	184 (97.87)	2854 (98.72)	620 (98.73)
Type 1	2 (0.12)	4 (2.13)	11 (0.38)	5 (0.80)
Type 2/unknown	11 (0.67)	0 (0.00)	26 (0.90)	3 (0.48)
Seizures	18 (1.10)	4 (2.13)	40 (1.38)	9 (1.43)
Epilepsy	10 (0.61)	2 (1.06)	28 (0.97)	7 (1.11)
MS characteristics				
Year of MS diagnosis (%)				
1945–1980			132 (4.57)	30 (4.78)
1981–1990			291 (10.07)	71 (11.31)
1991–2000			1122 (38.81)	265 (42.20)
2001–2005	138 (8.42)	17 (9.04)	1346 (46.56)	262 (41.72)
2006–2007	531 (32.42)	79 (42.02)		
2008–2009	438 (26.74)	61 (32.45)		
2010–2011	376 (22.95)	23 (12.23)		
2012–2014	155 (9.46)	8 (4.26)		
MS disease course (%)				
Relapsing remitting	1365 (83.33)	101 (53.72)	1878 (64.96)	244 (38.85)
Primary progressive	71 (4.33)	39 (20.74)	179 (6.19)	92 (14.65)
Secondary progressive	85 (5.19)	36 (19.15)	743 (25.70)	278 (44.27)
Unknown	117 (7.14)	12 (6.38)	91 (3.15)	14 (2.23)
Mean age at MS diagnosis, years (SD, min-max)	39.24 (0.26,18.02–64.82)	44.57 (0.73,19.93–64.71)	39.20 (0.20,18.04–64.88)	40.56 (0.43,18.09–64.86)
Onset N (% missing)	1464 (10.62)	172 (8.51)	2608 (9.79)	576 (8.28)
Mean age at MS onset, years (SD, min-max)	35.20 (0.27, 8.75–63.59)	38.57 (0.76, 15.54–62.29)	32.76 (0.19, 6.13–62.92)	33.62 (0.42, 11.75–62.46)
No of relapses N (% missing)	1007 (26.82)	323 (28.22)	2378 (17.74)	490 (21.97)
0 relapses (%)	104 (10.33)	29 (8.98)	77 (3.24)	15 (3.06)
1 relapse (%)	624 (61.97)	205 (63.47)	1400 (58.87)	299 (61.02)
2 relapses (%)	190 (18.87)	54 (16.72)	355 (14.93)	60 (12.24)
3 relapses (%)	55 (5.46)	22 (6.81)	213 (8.96)	44 (8.98)
4+ relapses (%)	34 (3.38)	13 (4.02)	333 (14.00)	72 (14.69)
First DMT N (% missing)	1198 (26.86)	132 (29.79)	2450 (15.25)	482 (23.25)
No DMT (%)	69 (4.80)	18 (13.04)	422 (17.22)	93 (19.29)
Moderately effective (%)	1195 (83.16)	104 (75.36)	1956 (79.84)	379 (78.63)
Highly effective (%)	173 (12.04)	16 (11.59)	72 (2.94)	10 (2.07)
Median years to first DMT, (IQR)	0.13 (0.05–0.36)	0.11 (0.04–0.26)	1.03 (0.18–4.90)	0.96 (0.13–5.03)
EDSS N (% missing)	739 (54.88)	75 (60.11)	1792 (38.01)	395 (37.10)
0 (%)	155 (20.97)	0 (0.00)	253 (14.12)	8 (2.03)
1–1.5 (%)	237 (32.07)	9 (12.00)	389 (21.71)	30 (7.59)
2–2.5 (%)	202 (27.33)	23 (30.67)	358 (19.98)	73 (18.48)
3–3.5 (%)	96 (12.99)	25 (33.33)	292 (16.29)	58 (14.68)
4–4.5 (%)	33 (4.47)	6 (8.00)	148 (8.26)	39 (9.87)
5–5.5 (%)	5 (0.68)	7 (9.33)	95 (5.30)	30 (7.59)
6–6.5 (%)	10 (1.35)	4 (5.33)	182 (10.16)	118 (29.87)
7–9.5 (%)	1 (0.14)	1 (1.33)	75 (4.19)	39 (9.87)

For many of the MS characteristics, there were missing values in each of the variables of interest. The percentage missing is listed in the table for each given variable.

*No individuals had a diagnosis of cerebral palsy or amyotrophic lateral sclerosis.

DMT, disease-modifying therapy; EDSS, Expanded Disability Severity Scale; max, maximum; min, minimum; MS, multiple sclerosis; N, total number of individuals; TBI, traumatic brain injury.

Median follow-up among pwIMS and pwPMS was 6.4 years (IQR= 4.5–7.9) and 8.5 years (IQR 8.5–8.5) respectively, with a median attained age of 45.5 years (IQR=37.7–53.0) and 55.7 years (IQR=48.2–64.4), respectively. Some 10.3% (95% CI 8.90% to 11.7%; n=188) of pwiMS received baclofen compared with 17.8% (95% CI 16.5% to 19.0%; n=628) of pwPMS. pwIMS and pwPMS who received baclofen were older at MS onset and diagnosis compared with those without baclofen. Most prescriptions occurred in recent calendar years, and within 6 months of MS diagnosis among pwIMS, and within 3 years of MS diagnosis among pwPMS. Approximately 30% of pwIMS and pwPMS received baclofen with an EDSS of 0–2.5. Incidence rate of baclofen initiation over the study period was 18 per 1000 people (95% CI 15.61 to 20.78) among pwIMS and 24 per 1000 (95% CI 21.70 to 25.37) among pwPMS.

### General risk factors

Among pwIMS, sex was associated with baclofen in all models (females compared with males HR 0.75 (95% CI 0.55 to 1.02)) (table 2). Disease course was strongly associated with baclofen, as progressive disease course had larger HRs compared with RRMS, even after adjustment for length of time with MS (PPMS HR 6.92, 95% CI 4.52 to 10.60; SPMS HR 5.57, 95% CI 3.82 to 8.13). A trend was seen with decreasing HRs with increasing MS time. No clear trend with year of MS diagnosis was observed, but HRs were elevated in all time periods. Age at MS onset showed no association.

**Table 2:** Individuals with incident MS and general variable associations with initiation of baclofen treatment

	Events	PT	M1		M2		M3		M4		M5		M6	
			HR	95% CI	HR	95% CI	HR	95% CI	HR	95% CI	HR	95% CI	HR	95% CI
Sex														
Male	72	2821	1.00		1.00		1.00		1.00		1.00		1.00	
Female	116	7618	0.63	0.47 to 0.85	0.64	0.47 to 0.86	0.75	0.55 to 1.02	0.73	0.53 to 1.00	0.75	0.55 to 1.02	0.76	0.56 to 1.03
Disease course														
RRMS	83	8286					1.00		1.00		1.00		1.00	
PPMS	39	509					7.17	4.69 to 10.97	6.95	4.44 to 10.87	6.92	4.52 to 10.60	7.00	4.58 to 10.70
SPMS	54	887					5.57	3.82 to 8.13	5.90	3.95 to 8.82	5.57	3.82 to 8.13	5.64	3.86 to 8.24
Unknown	12	756					1.64	0.89 to 3.03	1.28	0.55 to 2.95	1.63	0.88 to 3.01	1.63	0.88 to 3.00
Age at MS onset	172	9446							1.00	0.98 to 1.03				
Time with MS														
<0.5	20	799									1.00			
≥0.5 to <1	19	859									0.99	0.52 to 1.89		
≥1 to <2	28	1762									0.67	0.37 to 1.22		
≥2 to <3	26	1652									0.68	0.37 to 1.24		
≥3 to <6	68	3759									0.73	0.43 to 1.23		
≥6	27	1608									0.61	0.33 to 1.12		
Year of MS diagnosis														
2005	17	1232											1.00	
2006–2007	79	4471											1.17	0.69 to 2.00
2008–2009	61	2807											1.56	0.90 to 2.71
2010–2011	23	1558											1.24	0.66 to 2.36
2012–2014	8	371											1.81	0.76 to 4.33

Individuals with incident MS (N=1826) ever treated with baclofen (n=188) for their spasticity as the outcome. All models adjusted for age, county of residence at MS diagnosis and highest attainted educational level. Stratified Cox models used to account for non-proportionality in region of residence at MS diagnosis over time. Disease course and years with MS are time-varying covariates. Due to missing onset information (n=190), the model with age at onset includes N=1636 individuals.

The reference category is identified by an HR of 1.00 with no CI.

M, model; MS, multiple sclerosis; PPMS, primary progressive MS; PT, person time; RRMS, relapsing-remitting MS; SPMS, secondary progressive MS.

Similar trends were observed among pwPMS as above (table 3). Associations with disease course, though elevated among progressive patients, were of lower magnitude after adjustment for MS time than among pwIMS (PPMS HR 5.54, 95% CI 4.22 to 7.28; SPMS HR 4.40, 95% CI 3.60 to 5.38). Age at MS onset had a small magnitude effect (HR 1.01, 95% CI 1.00 to 1.02).

**Table 3:** Individuals with prevalent MS and general variable associations with initiation of baclofen treatment

	Events	PT	M1		M2		M3		M4		M5		M6		M7	
			HR	95% CI	HR	95% CI	HR	95% CI	HR	95% CI	HR	95% CI	HR	95% CI	HR	95% CI
Sex																
Male	207	6616	1.00		1.00		1.00		1.00		1.00		1.00		1.00	
Female	421	20 154	0.67	0.57 to 0.79	0.68	0.57 to 0.80	0.76	0.64 to 0.90	0.76	0.64 to 0.90	0.74	0.62 to 0.88	0.76	0.64 to 0.90	0.76	0.64 to 0.89
Disease course																
RRMS	179	15 730					1.00		1.00		1.00		1.00		1.00	
PPMS	92	1804					5.59	4.25 to 7.34	5.49	4.18 to 7.22	5.45	4.10 to 7.25	5.54	4.22 to 7.28	5.55	4.22 to 7.30
SPMS	343	8426					4.32	3.54 to 5.27	4.41	3.61 to 5.39	4.36	3.52 to 5.39	4.40	3.60 to 5.38	4.34	3.55 to 5.31
Unknown	14	811					1.71	0.99 to 2.96	1.67	0.97 to 2.89	1.69	0.79 to 3.63	1.67	0.97 to 2.89	1.70	0.98 to 2.94
Age at MS diagnosis	628	26 770							1.01	1.00 to 1.02						
MS-onset age	576	24 212									1.01	1.00 to 1.02				
Time with MS																
1 to <3	40	1822											1.00			
≥3 to <5	73	3230											0.89	0.48 to 1.63		
≥5 to <7	87	3979											0.87	0.49 to 1.54		
≥7 to <9	100	4130											0.82	0.46 to 1.44		
≥9 to <11	73	3430											0.88	0.50 to 1.55		
≥11 to <13	53	2352											0.74	0.42 to 1.32		
≥13 to <15	187	7201											0.74	0.41 to 1.34		
≥15+	30	1208											0.76	0.44 to 1.33		
Year of MS diagnosis																
1945–1980	71	2687													0.93	0.62 to 1.40
1981–1990	265	10 427													0.90	0.68 to 1.19
1991–2000	262	12 449													1.02	0.85 to 1.21
2001–2005	207	6616													1.00	

Individuals with prevalent MS (N=3519), including individuals ever treated for their spasticity using baclofen (n=628). All models adjusted for age, county of residence at MS diagnosis and highest attained educational level. Disease course and years with MS are time-varying covariates. Due to missing onset information (n=335), the model with age at onset includes N=3184 individuals. Reference categories are indicated by an HR of 1.00 with no CI.

M, model; MS, multiple sclerosis; PPMS, primary progressive MS; PT, person time; RRMS, relapsing-remitting MS; SPMS, secondary progressive MS.

### Clinical MS variables

Among pwIMS, EDSS at baseline was associated with baclofen treatment, with increasing HRs with increasing EDSS (table 4, panel A). After controlling for other MS characteristics the associations remained: EDSS 3.0–5.5, HR 3.05, 95% CI 1.61 to 5.76; EDSS 6+, HR 2.74, 95% CI 0.51 to 14.65. At MS diagnosis, both disease course and EDSS were strong predictors of baclofen initiation, although EDSS was the stronger predictor. However, when EDSS was allowed to vary over time, age-specific patterns emerged with larger magnitude HRs especially among younger pwIMS (table 4, panel B). Individuals aged 18–44 years with EDSS 3.0–5.5 compared with same-aged individuals with EDSS 0–2.5 had an HR of 5.62 (95% CI 2.91 to 10.85). HRs increased with increasing disability: EDSS 6+, HR 15.41, 95% CI 7.07 to 33.58 even after controlling for time with MS, DMTs and disease course. At ages 45–73 years people with EDSS 3.0–5.5 or 6+ compared with EDSS 0–2.5, HRs were still elevated, but much smaller in magnitude (HR 2.05, 95% CI 1.10 to 3.82; HR 4.26, 95% CI 1.96 to 9.17, respectively). Thus, individuals with the same EDSS score, but at different ages had differences in the magnitude of the association with baclofen initiation, irrespective of when EDSS was measured. Sex differences observed in general models disappeared in these analyses when controlling for MS-specific characteristics. DMTs showed increased HRs, although few individuals used highly effective DMTs.

**Table 4:** Incident MS—Association of Expanded Disability Severity Scale (EDSS) scores as a baseline and time-varying variable and other MS characteristics to baclofen treatment

Panel A: baseline EDSS	E		M1		M2		M3		M4		M5		M6		M7	
		PT	HR	95% CI	HR	95% CI	HR	95% CI	HR	95% CI	HR	95% CI	HR	95% CI	HR	95% CI
EDSS																
EDSS 0–2.5	32	3711	1.00		1.00		1.00		1.00		1.00		1.00		1.00	
EDSS 3–5.5	38	895	3.85	2.35 to 6.31	3.78	2.29 to 6.26	3.50	2.09 to 5.84	2.89	1.70 to 4.93	3.05	1.61 to 5.76	3.13	1.69 to 5.78	3.07	1.62 to 5.80
EDSS 6+	5	75	4.66	1.73 to 12.59	4.33	1.59 to 11.80	3.83	1.38 to 10.61	2.94	1.06 to 8.16	2.74	0.51 to 14.65	2.99	0.62 to 14.47	2.49	0.46 to 13.58
Sex																
Male	27	1239	1.00		1.00		1.00		1.00		1.00		1.00		1.00	
Female	48	3442	0.83	0.51 to 1.35	0.84	0.51 to 1.38	0.83	0.50 to 1.37	0.86	0.52 to 1.43	1.15	0.61 to 2.16	1.09	0.59 to 2.04	1.15	0.61 to 2.16
Age at MS onset	72	4447			1.02	0.99 to 1.05	1.02	0.99 to 1.05	1.03	0.99 to 1.07	1.02	0.98 to 1.06	1.01	0.97 to 1.04	1.02	0.98 to 1.07
Course at entry																
RRMS	38	3912							1.00		1.00				1.00	
PPMS	14	180							4.29	2.05 to 8.98	3.41	1.11 to 10.47			3.37	1.09 to 10.45
SPMS	20	345							5.14	2.64 to 9.98	5.86	2.65 to 12.98			6.24	2.80 to 13.93
Unknown	3	243							1.03	0.24 to 4.36	2.03	0.46 to 8.93			2.18	0.49 to 9.61
DMT use																
No DMT	9	546									1.00		1.00		1.00	
Moderately effective	36	3162									1.48	0.60 to 3.67	0.89	0.39 to 2.00	1.39	0.54 to 3.57
Highly effective	9	434									1.19	0.40 to 3.56	0.94	0.32 to 2.75	1.13	0.36 to 3.53

Panel B: time-varying EDSS	E		M1		M2		M3		M4		M5		M6		M7	
		PT	HR	95% CI	HR	95% CI	HR	95% CI	HR	95% CI	HR	95% CI	HR	95% CI	HR	95% CI
EDSS																
0–2.5 age 18–44	22	4479	1.00		1.00		1.00		1.00		1.00		1.00		1.00	
0–2.5 age 45–73	23	2196	1.00		1.00		1.00		1.00		1.00		1.00		1.00	
3–5.5 age 18–44	21	701	5.33	2.88 to 9.87	5.91	3.20 to 10.92	5.90	3.22 to 10.83	5.59	3.06 to 10.20	5.49	2.86 to 10.53	5.60	2.92 to 10.73	5.62	2.91 to 10.85
3–5.5 age 45–73	35	1131	2.76	1.62 to 4.71	2.47	1.44 to 4.23	2.38	1.38 to 4.10	1.99	1.14 to 3.48	2.07	1.11 to 3.84	2.31	1.25 to 4.26	2.05	1.10 to 3.82
6+age 18–44	13	118	18.83	9.61 to 36.88	20.66	10.18 to 41.95	23.66	11.88 to 47.15	17.84	9.32 to 34.15	15.61	7.22 to 33.75	18.28	8.21 to 40.67	15.41	7.07 to 33.58
6+age 45–73	25	244	9.31	5.15 to 16.82	8.13	4.45 to 14.85	7.76	4.24 to 14.22	5.13	2.76 to 9.52	4.27	1.99 to 9.18	6.29	3.03 to 13.05	4.26	1.97 to 9.17
Sex																
Male	50	2334	1.00		1.00		1.00		1.00		1.00		1.00		1.00	1.00
Female	89	6535	0.82	0.58 to 1.17	0.79	0.56 to 1.13	0.81	0.57 to 1.15	0.86	0.61 to 1.23	0.90	0.60 to 1.33	0.96	0.64 to 1.44	0.94	0.62 to 1.42
Age at MS onset	132	8264			1.00	0.98 to 1.02	1.00	0.98 to 1.02	1.01	0.99 to 1.03	1.01	0.98 to 1.03	1.00	0.98 to 1.02	1.01	0.98 to 1.04
Disease course																
RRMS	80	7450							1.00		1.00				1.00	
PPMS	27	411							2.73	1.67 to 4.47	2.91	1.56 to 5.44			2.88	1.53 to 5.43
SPMS	29	604							2.79	1.66 to 4.69	2.59	1.39 to 4.81			2.66	1.43 to 4.95
Unknown	3	403							0.59	0.16 to 2.15	0.81	0.21 to 3.09			0.80	0.20 to 3.15
DMT use																
No DMT	17	1095									1.00		1.00		1.00	
Moderately effective	79	6263									1.31	0.75 to 2.28	0.92	0.53 to 1.59	1.20	0.67 to 2.16
Highly effective	13	672									1.32	0.60 to 2.91	1.00	0.46 to 2.19	1.20	0.52 to 2.77

**Reference categories indicated by HRs of 1.00 with no 95% CI. Panel A:** Association of baseline EDSS to baclofen treatment. Model 1 adjusted for age, county of residence at MS diagnosis and highest attained education. Model 3 additionally adjusted for calendar year of MS diagnosis. Model 7 additionally adjusted for time with MS as an additional timescale. Disease-modifying treatment is a time-varying variable. Due to missing values, the number of individuals vary. Model 1 N=814, models 2–4 N=767, models 5–7 N=672.

**Panel B**: Association of time-varying EDSS to baclofen. Due to violation of the proportional hazard assumption, an interaction between age (timescale) and EDSS was used. Reference categories are the lowest EDSS category at each respective age group as compared with the next EDSS level in that specific age group. Model 1 adjusted for age, county of residence at MS diagnosis and highest attained education. Model 3 additionally adjusted for calendar year of MS diagnosis. Model 7 additionally adjusted for time with MS as an additional timescale. Due to missing values, the number of individuals vary: model 1 N=1514, models 2–4 N=1402 and models 5–7 N=1261. Disease-modifying treatments is a time-varying variable. Robust SE used to calculate confidence intervals due to repeated measurements of EDSS.

DMT, disease-modifying therapy; E, number of events; HR, Hazard ratio; M, model; PPMS, primary progressive MS; PT, person time; RRMS, relapsing remitting MS; SPMS, secondary progressive MS.

Relapses showed no association with baclofen initiation among relapsing-remitting pwIMS (online supplemental table 2).

10.1136/jnnp-2022-329886.supp3 Supplementary data

Similar HRs were observed among pwPMS for EDSS at baseline, but the magnitudes of the associations with baclofen initiation were somewhat reduced (table 5, panel A). EDSS as a time-varying covariate also showed age-specific associations as seen among the incident MS cohort, and EDSS had higher magnitude associations than disease course to baclofen initiation (table 5, panel B).

**Table 5:** Prevalent MS—Association of Expanded Disability Severity Scale (EDSS) scores as a baseline and time-varying variable and other MS specific to baclofen treatment

Panel A: baseline EDSS	E	PT	M1		M2		M3		M4		M5		M6		M7	
			HR	95% CI	HR	95% CI	HR	95% CI	HR	95% CI	HR	95% CI	HR	95% CI	HR	95% CI
EDSS																
EDSS 0–2.5	111	8978	1.00		1.00		1.00		1.00		1.00		1.00		1.00	
EDSS 3–5.5	127	4995	2.30	1.77 to 3.00	2.32	1.78 to 3.03	2.31	1.77 to 3.02	1.87	1.40 to 2.48	1.86	1.37 to 2.52	2.19	1.64 to 2.92	1.86	1.37 to 2.52
EDSS 6+	157	2647	6.00	4.57 to 7.86	6.15	4.68 to 8.09	6.12	4.64 to 8.06	3.92	2.83 to 5.44	3.85	2.66 to 5.56	5.62	4.11 to 7.67	3.82	2.65 to 5.53
Sex																
Male	126	4226	1.00		1.00		1.00		1.00		1.00		1.00		1.00	
Female	269	12 394	0.77	0.62 to 0.95	0.78	0.63 to 0.96	0.78	0.63 to 0.96	0.81	0.65 to 1.00	0.87	0.68 to 1.11	0.84	0.66 to 1.07	0.87	0.68 to 1.10
Age at MS diagnosis	395	16 620			1.01	1.00 to 1.03			1.00	0.97 to 1.04	1.01	0.98 to 1.04	1.01	0.98 to 1.05	1.03	0.97 to 1.09
Disease course																
RRMS	148	10 639							1.00		1.00				1.00	
PPMS	52	1023							2.34	1.60 to 3.42	2.49	1.58 to 3.93			2.48	1.57 to 3.92
SPMS	192	4813							1.94	1.46 to 2.59	1.70	1.25 to 2.33			1.70	1.24 to 2.32
Unknown	3	146							1.17	0.37 to 3.73	1.51	0.47 to 4.87			1.52	0.47 to 4.90
DMT use																
No DMT	55	2870									1.00		1.00		1.00	
Moderately effective	262	11 703									1.52	1.10 to 2.09	1.35	0.99 to 1.84	1.53	1.11 to 2.10
Highly effective	4	177									1.47	0.52 to 4.15	1.26	0.45 to 3.56	1.47	0.52 to 4.17

Panel B: Time-varying EDSS	E	PT	M1		M2		M3		M4		M5		M6		M7	
			HR	95% CI	HR	95% CI	HR	95% CI	HR	95% CI	HR	95% CI	HR	95% CI	HR	95% CI
EDSS																
0–2.5 age 19–49	40	7090	1.00		1.00		1.00		1.00		1.00		1.00		1.00	
0–2.5 age 50–92	45	4328	1.00		1.00		1.00		1.00		1.00		1.00		1.00	
3–5.5 age 19–49	81	2906	5.19	3.54 to 7.61	5.25	3.58 to 7.70	5.21	3.55 to 7.65	4.91	3.34 to 7.22	4.97	3.34 to 7.40	5.19	3.50 to 7.71	4.95	3.32 to 7.36
3–5.5 age 50–92	95	4642	2.04	1.43,2.92	2.05	1.44 to 2.93	2.05	1.44 to 2.93	1.74	1.20 to 2.52	1.59	1.06 to 2.40	1.77	1.19 to 2.63	1.59	1.06 to 2.39
6+ age 19–49	72	875	15.15	10.18,22.55	15.51	10.42 to 23.11	15.38	10.31 to 22.94	12.52	8.21 to 19.08	12.35	7.89 to 19.32	14.00	9.12 to 21.49	12.26	7.83 to 19.20
6+ age 50–92	173	3938	4.75	3.39 to 6.68	4.85	3.45 to 6.82	4.83	3.44 to 6.79	3.68	2.55 to 5.32	3.49	2.31 to 5.28	4.26	2.91 to 6.23	3.46	2.29 to 5.24
Sex																
Male	167	5922	1.00		1.00		1.00		1.00		1.00		1.00		1.00	
Female	339	17 856	0.72	0.59 to 0.86	0.72	0.60 to 0.87	0.72	0.59 to 0.86	0.74	0.62 to 0.90	0.79	0.64 to 0.97	0.77	0.62 to 0.95	0.79	0.64 to 0.97
Age at MS diagnosis	506	23 778			1.01	1.00 to 1.02			1.01	0.98 to 1.04	1.01	0.98 to 1.04	1.01	0.98 to 1.04	1.04	0.99 to 1.10
Course																
RRMS	202	15 382							1.00		1.00				1.00	
PPMS	69	1517							1.81	1.33 to 2.47	1.77	1.20 to 2.61			1.78	1.21 to 2.63
SPMS	232	6565							1.46	1.15 to 1.84	1.26	0.98 to 1.64			1.26	0.98 to 1.64
Unknown	3	314							0.50	0.16 to 1.58	0.68	0.22 to 2.16			0.68	0.22 to 2.16
DMT use																
No DMT	76	4328									1.00		1.00		1.00	
Moderately effective	324	16 290									1.46	1.11 to 1.93	1.33	1.02 to 1.75	1.46	1.11 to 1.93
Highly effective	7	351									1.23	0.55 to 2.72	1.08	0.49 to 2.37	1.22	0.55 to 2.71

**Reference categories indicated by HRs of 1.00 with no 95% CI. Panel A:** Association of baseline EDSS to baclofen treatment. Model 1 adjusted for age, county of residence at MS diagnosis, and highest attained education. Model 3 additionally adjusted for calendar year of MS diagnosis. Model 7 additionally adjusted for time with MS as an additional timescale. Disease-modifying treatments is a time-varying variable. Note, due to missing values, the number of individuals in each model vary. Model 1–4 N=2187, models 5–7 N=1917.

**Panel B:** Association of time-varying EDSS to spasticity treatment. Due to violation of the proportional hazard assumption, an interaction between age and EDSS was used. Model 1 adjusted for age, county of residence at MS diagnosis, and highest attained education. Model 3 additionally adjusted for calendar year of MS diagnosis. Model 7 additionally adjusted for time with MS as an additional timescale. Disease-modifying treatment is a time-varying variable. Robust SE used to calculate CIs due to repeated measurements of EDSS. Note, due to missing values, the number of individuals in each model vary. Models 1–4 N=2187, models 5–7 N=1917.

DMT, disease-modifying therapy; E, number of events; HR, Hazard ratio; M, model; MS, multiple sclerosis; PPMS, primary progressive MS; PT, person time; RRMS, relapsing remitting MS; SPMS, secondary progressive MS.

### Sensitivity analyses

Widening the definition of spasticity treatments (secondary outcome) showed that baclofen and gabapentin were the most commonly prescribed (online supplemental table 1). Similar treatment patterns were found as in the main analyses among both pwIMS and pwPMS, but the magnitude of all associations reduced, especially for general characteristics (online supplemental tables 3 and 4) and EDSS scores (online supplemental table 9). Increased HRs were observed even at low EDSS values (0–3.0) as compared with no EDSS score. No association with sex was observed. After further excluding pwIMS or pwPMS diagnosed with seizures (online supplemental tables 5 and 7) or individuals treated/diagnosed with depression (online supplemental tables 6 and 8) the results were similar to the main analysis although with somewhat reduced HRs.

10.1136/jnnp-2022-329886.supp2 Supplementary data

10.1136/jnnp-2022-329886.supp4 Supplementary data

### Comorbid diseases as risk factors

Associations of comorbid diseases with baclofen initiation as a primary outcome could not be determined due to small numbers, but were assessed using the secondary outcome. All associations (except with depression) should be interpreted with caution due to small numbers and wide confidence intervals. In general, a diagnosis/being treated for a comorbid disease among both pwIMS and pwPMS showed increased HRs for all possible spasticity treatments, though the majority of HRs were of larger magnitude among pwIMS (online supplemental table 10). Depression, seizures and Parkinson’s disease had the largest magnitude HRs. Stroke showed no association among pwIMS, but risk factors for stroke (vascular disease and coronary artery disease) had increased HRs for baclofen initiation. Among pwPMS, seizures, depression and concussion had increased HRs for baclofen initiation.

10.1136/jnnp-2022-329886.supp5 Supplementary data

### Baclofen discontinuation

**Figure 3:**
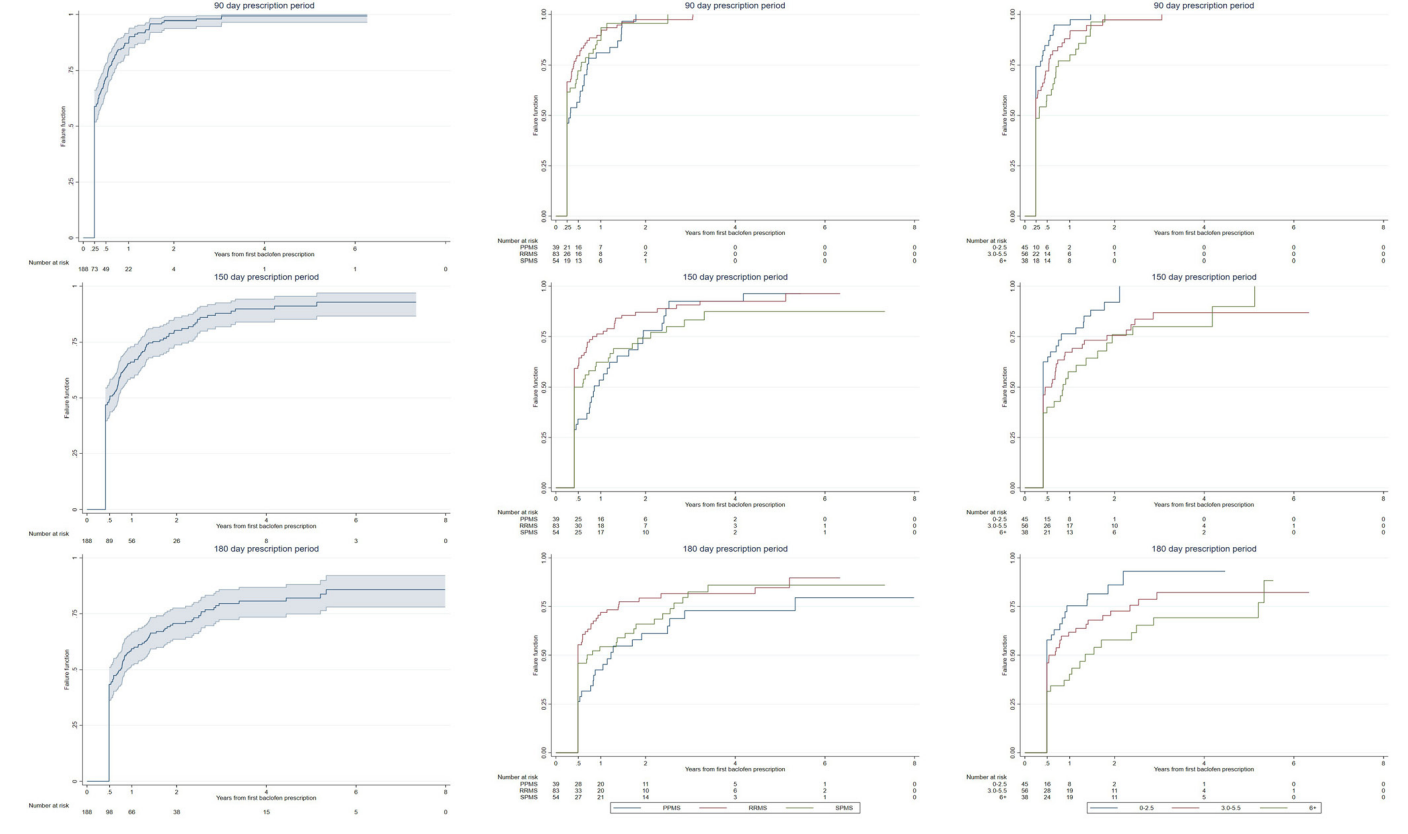
Time to discontinuation of baclofen among people with incident multiple sclerosis stratified by disease course and Expanded Disability Severity Scale (EDSS). Left column, overall discontinuation. Middle column, discontinuation stratified by disease course when starting baclofen. Right column, discontinuation stratified by EDSS score when starting baclofen. PPMS, primary progressive multiple sclerosis; RRMS, relapsing-remitting multiple sclerosis; SPMS, secondary progressive multiple sclerosis.

Discontinuation rates of baclofen among pwIMS (figure 3) and pwPMS (online supplemental figure 1) were similar and showed that irrespective of the gap period used (90, 150 or 180 days), 50% of individuals discontinued rapidly and 90% discontinued overall. Among pwIMS and pwPMS and using a gap period of 150 days between prescriptions, 65% discontinued within the first year. Differences between pwIMS and pwPMS were only evident when stratifying by disease course as pwIMS with progressive courses continued longer than RRMS, though among pwPMS the differences in discontinuation between disease courses were less clear. Stratification by EDSS in either the pwIMS or pwPMS cohorts showed individuals of higher EDSS persisting longer on baclofen.

10.1136/jnnp-2022-329886.supp6 Supplementary data

#### Discontinuation of other spasticity treatments

Discontinuation of gabapentin, diazepam or baclofen among pwIMS (whichever was prescribed first) also occurred rapidly after initiation (online supplemental figures 2–4). Gabapentin was discontinued by 75% of individuals after 1 year, and diazepam nearly completely discontinued after 6 months, and identically as the main analyses for baclofen. Too few individuals received clonazepam or cannabinoids to determine their discontinuation.

10.1136/jnnp-2022-329886.supp7 Supplementary data

10.1136/jnnp-2022-329886.supp8 Supplementary data

10.1136/jnnp-2022-329886.supp9 Supplementary data

## Discussion

To the best of our knowledge, this is the first study consisting of a real-world MS population followed for a period of 10 years capturing routine clinical practice in treating spasticity among both incident and prevalent patients with MS. Baclofen remains the first-line option for managing spasticity in MS, and patterns of initiation and discontinuation described in this study are relevant to inform future clinical practice. Newly diagnosed pwMS and individuals with MS for many years can receive treatment for spasticity. MS is the likely cause for requiring baclofen as no other disease known to cause spasticity is associated with baclofen. EDSS measured any time after MS diagnosis is a strong predictor of baclofen prescription, especially among younger patients and this was irrespective of changes to EDSS. Younger compared with older patients with MS of similar disability showed different patterns of spasticity treatment as younger patients were more likely to start baclofen. All associations were observed even after finely controlling for time with MS, demonstrating that although MS severity tends to increase over time, it is disability and likely motor symptoms that contribute to the need for baclofen. Progressive disease course was also highly associated with baclofen treatment, first only among males, but these sex differences were eliminated after adjustment for MS characteristics. Baclofen initiation among pwIMS is highest within 6 months from diagnosis, and among pwPMS, within the first 3 years after diagnosis although this may reflect patients with prevalent disease reinitiating baclofen use after an initial rapid discontinuation as seen in this study. The vast majority of individuals treated with baclofen rapidly discontinued use, with more than 50% discontinuing within 6 months. This high rate of discontinuation even among more disabled patients and those with progressive disease demonstrates the lack of efficacy or tolerability of baclofen and other spasticity treatments, in turn leading to a low success rate of treating and managing spasticity with current pharmacological agents.

There were indications that even at low EDSS levels spasticity may affect pwMS, particularly at younger ages. Some 30% of baclofen prescriptions were given to both pwIMS and pwPMS with EDSS scores less than three. However, as we are measuring treatment rather than spasticity directly, prescriptions may be to treat pain mistaken for spasticity, spasticity at a very low severity or to treat suspected spasticity resulting in discontinuation after no improvement or due to side effects. EDSS measurement error may also be a factor, as updates to EDSS can be delayed, or are not updated. Given the wide range of symptoms of spasticity, increases in muscular tone may not be the first indications of spasticity and as MS progresses, spasticity may manifest more apparently at a clinically significantly detectible level. Nonetheless, this highlights the importance of early identification and diagnosis of spasticity among pwMS given we observed an association with increasing age at MS onset and baclofen initiation, in order to advise on non-pharmacological treatments at very mild levels of spasticity or pain to reduce debilitating symptoms prior to needing baclofen given its side effects and need for it at a more severe level of disability. Cannabinoids represents a more recent pharmacological treatment option for MS-associated spasticity, where an oral preparation of delta-9-tetrahydrocannabinol has been approved by the European Medicines Agency. However, its use in Sweden is very limited and it is not included in the national drug reimbursement programme. In our study, only four patients have been prescribed this type of cannabinoid. Nevertheless, further studies in other cohorts should address whether the tolerability profile of cannabinoids is superior to that of current first-line and second-line pharmacological treatments.

Previous studies have largely suggested that spasticity is more common among people who have had MS for a longer period of time, among males and especially among individuals with a progressive course.^4^ This was also true here; however, sex differences disappeared after adjusting for MS characteristics. We also show that a large proportion of individuals receiving baclofen have RRMS and that pwMS require spasticity treatment early after MS diagnoses and at younger ages. Progressive disease courses showed higher magnitude associations with baclofen treatment, which is unsurprising given the increasing motor symptoms and disability accompanying progressive MS. However, the occurrence of spasticity indicated by treatment in our study is much lower than self-reported prevalence in another study.^22^ This may reflect us capturing more severe spasticity requiring baclofen as well as we do not capture non-prescription treated spasticity. At lower levels of spasticity severity, treatments can be both over-the-counter or non-pharmacological until spasticity can no longer be managed,^23 24^ something we are unable to capture in this study. It is also possible that other studies where spasticity is self-reported, PwMS may attribute a wider range of symptoms to spasticity, while treating neurologists rely more on objective findings such as increased muscle tone. We believe that no other study has directly examined the time to rapid discontinuation of baclofen as we assessed in our study, but other studies discuss discontinuation. Discontinuation is likely due to the low tolerability of the treatments given their wide-range of side effects such as dizziness or low blood pressure.^25 26^

Broadening the definition of spasticity treatments attenuated the magnitude of associations observed with baclofen. However, baclofen may not be the first choice for spasticity treatment especially among individuals with RRMS. Treatments such as gabapentin and diazepam can be prescribed for spasticity as shown in other studies^4 6^ although we cannot be sure of the reason for their prescription in our study. Although they are primarily indicated for treatment of neuropathic pain, gabapentin has been shown to effectively relieve spasticity and spasticity-related pain.^8^ Not all patients are able to be treated with baclofen, as drug–drug interactions can occur between baclofen, tricyclic antidepressants and antihypertensives.

Studies among general populations have shown that stroke, head trauma and other diseases increased the risk of spasticity,^5 27 28^ although this could not be verified in this MS population. This instead points to MS-specific spasticity due to pathological changes caused by MS. pwIMS overall had larger magnitude associations if they had comorbid diseases compared with pwPMS, but pwPMS were much older: age could be the more important factor that reduces the associations as older adults experience a larger comorbid disease burden in general,^29^ reducing differences among treatment groups. The results could also be explained by an increased number of contacts with healthcare increasing the probability of receiving a treatment for spasticity, however it is likely an actual increase in spasticity for those with additional comorbid diseases as results are similar among both pwIMS and pwPMS. Additionally, the age-related differences we observed between younger and older similarly disabled patients could be due to an increased sensitivity to side effects among older patients, or that older patients may have a greater perceived or real risk of falling when treating for spasticity as spasticity-associated muscle rigidity can provide a degree of postural stability. Collectively, this may lead to reduced tolerability compared with younger individuals with similar disability level.

### Advantages and disadvantages

This large, register-based study is the first to our knowledge to include both incident and prevalent MS cohorts. Baclofen treatment was not subject to recall bias or lost to follow-up as all individuals were followed throughout the study period. We examined a wide variety of possible MS characteristics, comorbid diseases and individual factors, which were not subject to types of bias found in self-reported studies. Although we were not able to confirm if individuals had spasticity when receiving baclofen, baclofen is used almost exclusively to treat spasticity in patients with MS in Sweden. The only exception being that baclofen is also considered a second-line option in guidelines from the Swedish Medical Products Agency for painful Lhermitte and trigeminal neuralgia (together with gabapentin, lamotrigine and phenytoin),^10^ two conditions known to affect only a minority of patients with MS. We also broadened the definition of spasticity treatment to other pharmacological treatments that have broader indications including pain, and performed multiple sensitivity analyses to exclude coindicated diseases and counter-indicated prescriptions that confirmed the main results and ensured a good approximation of spasticity by using treatment as a proxy for spasticity. The severity of spasticity could not be captured, however we likely captured spasticity that is more severe in nature especially among pwPMS, but very severe spasticity was likely not captured here as intrathecal baclofen pumps, Botox injections and second-line treatments were not identified. These are not usually a first treatment choice for spasticity over oral baclofen or the other preparations used to identify spasticity in this study. Nevertheless, in both cohorts, spasticity is likely underestimated rather than overestimated as over-the-counter medications for pain and non-pharmacological treatments are not captured. Among the prevalent population in particular, we may be underestimating spasticity as individuals prior to 2005 (when the PDR began) could have had an initial spasticity treatment that we cannot observe, however, they were still eligible to receive another prescription during the study period.

Associations of specific DMTs with baclofen could not be explored in this study, as most individuals received interferon treatments or glatiramer acetate. The study period did not cover the years in which more highly effective DMTs became more commonly prescribed rather than reserved for individuals with more severe MS or as escalation therapies. The individuals most likely to receive highly effective DMTs had more severe MS and thus were more likely to have spasticity, which largely explains the increased HRs of DMT with baclofen initiation.

## Conclusion

pwMS can require treatment for spasticity even early post-MS diagnosis, at lower levels of MS disability and at young ages. High rates of discontinuation of treatment highlights an unmet need of tolerable and effective spasticity treatment alternatives. Taken together, this further highlights the importance of informed treatment options and better understanding of spasticity in general among patients with MS.

## Data Availability

Data are available on reasonable request.
