# Supplementary material for: Spasticity treatment patterns among people with multiple sclerosis: a Swedish cohort study
Source: J Neurol Neurosurg Psychiatry. 2022 Dec 20;94(5):337–48. doi: 10.1136/jnnp-2022-329886 (PMC10176386; doi:10.1136/jnnp-2022-329886)
Supplement: Supplementary data [file jnnp-2022-329886supp001.pdf]

Supplementary Table 0: ICD-10 codes used to identify diseases from the National Patient Register and Anatomical Therapeutic Chemical (ATC) codes used to identify prescriptions from the Prescribed Drug Register to classify individuals with a specific disease.

**Disease category**	**National Patient Register ICD-10 diagnoses.** Codes in **bold** were extracted from the register.	**Prescribed Drug Register ATC prescription codes.** Codes in **bold** were extracted from the register.	**Drug name**	**How individuals were identified with the disease**	**Reason for disease inclusion**
**Amyotrophic lateral sclerosis (ALS)**	**G12.2**			The first diagnosis of ALS within the National Patient Register was used as the date of diagnosis of ALS. Given the speed as to which ALS progresses, individuals were also identified from the Cause of Death register using the same ICD-10 code.	Individuals with either or ALS and cerebral palsy have been shown to have spasticity.
**Cardiovascular diseases and disorders** [1]	**Vascular hypertensive disorders: I10-I13, I15**<br>**Coronary artery disorders: I20-I25**			First recorded diagnosis of either disease was used as the date of vascular hypertension, or coronary artery disease.	Vascular hypertensive disorders and coronary artery disorders are risk factors for stroke. Individuals who have experience a stroke are more likely to have spasticity.
**Cerebral palsy**	**G80**			The earliest date of diagnosis in the National Patient Register was used to identify individuals with cerebral palsy.	Individuals with cerebral palsy are known to have spasticity.
**Depression**[2]<br><br>NOTE: the drug names are the ones currently recommended for use in Sweden under treatment guidelines. The ATC prescription categories contain more ATC codes, with drugs used previously. Both	**F31 – Bipolar affective disorder**<br><br><br>**F32: Depressive episode**<br>F32.0 – Mild depressive symptom<br>F32.1 – Moderate depression<br>F32.2- Severe without psychotic episode<br>F32.3- Severe with psychotic episode<br>F32.8 – Other depressive episodes<br>F32.9 – Depressive episode, unspecified<br><br>**F33: Recurrent depressive episode**<br>**F33.0**— Mild	**'N06A' +(A-G,X)**<br>**TCA:** 'N06AC'<br><br><br>N06AA09<br>N06AA04<br>N06AA21, 'N06AC01<br>N06AA10<br><br><br>**SSRI:** 'N06AB'<br><br>N06AB04<br>N06AB10<br>N06AB03<br>N06AB08<br>N06AB05<br>N06AB06	**Antidepressants**<br>**TCA: (Tricyclic antidepressants)/Non-specific monoamine oxidase inhibitors**<br>Amitriptyline<br>Clomipramine<br>Maprotiline<br>Nortriptyline<br><br><br>**SSRI: (Selective serotonin reuptake inhibitors)**<br>Citalopram<br>Citalopram<br>Fluoxetine<br>Fluvoxamine<br>Paroxetine<br>Sertraline	First ever date of prescription or of diagnosis used to identify depression. Earliest date of prescription or diagnosis used as the date of the start of depression.	Some of the spasticity treatments are also indicated for anxiety and pain, among other diseases/conditions. Therefore, it is important to identify individuals with depression to determine the overlap between depression and spasticity treatments/diagnoses. Depression can also increase pain, a symptom that is also associated with spasticity.<br><br>Some spasticity treatments, such as baclofen, are counter indicated for use with tricyclic antidepressants, which may lead to use of other spasticity

[1] Treatment of cardiovascular diseases in Sweden: https://www.lakemedelsverket.se/globalassets/dokument/behandling-och-forskrivning/behandlingsrekommendationer/behandlingsrekommendation/behandlingsrekommendation-att-forebygga-aterosklerotisk-hjart-karlsjukdom-med--lakemedel.pdf

[22] Treatment of depression in Sweden: https://www.lakemedelsverket.se/globalassets/dokument/behandling-och-forskrivning/behandlingsrekommendationer/behandlingsrekommendation/behandlingsrekommendation-lakemedel-depression-angestsyndrom-tvangssyndrom.pdf

are included for completeness.	**F33.1** – moderate<br>**F33.2** – severe with psychotic<br>**F33.3** – severe without psychotic<br>**F33.8** – other<br>**F33.9** – unspecified<br><br>**F34 – Persistent mood disorders**<br>**F38**—Other mood disorders<br>**F39** – Unspecified mood disorder	**MAOI: '**N06AF', 'N06AG'<br><br>N06AG02<br><br>**Other: 'N06AX'**<br><br>N06AX03<br>N06AX11<br><br>**Other: 'N06AX'**<br><br>N06AX21<br>N06AX16<br><br>N06AX12<br>N06AX18<br><br>N06AX22<br><br>N06AX26	**MAOI: (Monoamine oxidase inhibitor)**<br>Moclobemide<br><br>**NaSSA: (Noradrenergic and specific serotonergic antidepressants)**<br>Mianserin<br>Mirtazapine<br><br>**SNRI: (Serotonin norepinephrine reuptake inhibitors)[3]**<br>Duloxetine<br>Venlafaxine<br><br>**Other:**<br>Bupropion<br>Reboxetine (Noradrenergic reuptake inhibitors)<br>Agomelatine (limited use in Sweden)<br>Vortioxetine (limited use in Sweden)		pharmacological treatments instead of baclofen.
**Diabetes**	**E10** – Type I diabetes<br>**E11**—Type II diabetes<br>**E13** – Other specified diabetes<br>**E14** – Non-specific diabetes			Individuals with ICD-10 codes of E13 and E14 combined together into "unknown" diabetes category. In the event of multiple diagnoses, the earliest date of diabetes diagnosis was used.	Diabetes is a risk factor for both cardiovascular diseases and stroke, both possibly leading to an increased risk in spasticity.
**Epilepsy Seizures** [4]	G40<br>G41	Anti-epileptics were not available.		Individuals with at least two diagnoses of seizure/epilepsy were used to identify individuals with epilepsy, as there is considerable overlap between both ICD-10 codes.	Seizures among people with MS have been previously shown be associated with spasticity.
**Stroke/TIA**	**Ischemic stroke**: I63, I64, I69*, G46**<br>**Haemorrhagic stroke**: I60, I61, I62, I69*, G46**<br>**TIA:** G45			Due to the limited number of individuals with stroke, all types of stroke were combined, and classified as ever vs never stroke. In the event of multiple diagnoses,	Individuals who have experienced a stroke are also likely to develop spasticity.

[3] For treatment of severe depression, SNRI (mainly venlafaxine) has been shown to be more effective than SSRIs.

[4] Treatment of seizures and epilepsy in Sweden: https://www.lakemedelsverket.se/globalassets/dokument/behandling-och-forskrivning/behandlingsrekommendationer/behandlingsrekommendation/behandlingsrekommendation-epilepsi.pdf

	*I69 includes sequelae of both ischemic and haemorrhagic stroke<br>**Vascular syndromes of brain in cerebrovascular diseases could be both ischemic and haemorrhagic.			the first diagnosis was used as the date of stroke.	
**Traumatic brain injuries (TBI) causing bleeding due to external causes.**	S06.1, S06.3-S06.8*<br>*All S chapter ICD codes are traumatic brain injury types of bleeding most similar to stroke, excluding concussion. Not included under stroke as these are due to external causes.			Concussion and TBI (first date of either) are combined into one variable as they were a rare occurrence among individuals in this study.	Concussions and TBI may be associated with spasticity.
**Concussion & spinal cord injury**	S06.0 - Concussion<br>S06.2 – Diffuse brain injury<br>S06.9 – Intracranial injury, unspecified<br>T90.5 – sequelae of intracranial injury<br><br>**Spinal cord injuries**<br>T02.0<br>T02.1<br>T02.7<br>T03.0<br>T03.1<br>T04.0<br>T04.1<br>T06.0<br>T06.1<br>T08<br>T09.3			Concussion and TBI (first date of either) are combined into one variable as they were a rare occurrence among individuals in this study.<br><br>**Note:** No one in the dataset had any of the spinal cord injury ICD codes. One person had a 4th diagnosis of one of the codes, but likely a suspected spinal cord injury. Not include in further analyses.	Concussions and TBI may be associated with spasticity.
**Parkinson's disease**	G20 – Primary Parkinson's disease<br>G21 – Secondary parkinsonism				Due to similar clinical presentation of tremors, rigidity and overlap with respects to possible mechanisms causing Parkinson's symptoms and Parkinson's like symptoms, it is possible that an individual receives an ICD code in hospital for Parkinson's disease.
