# Supplementary material for: Spasticity treatment patterns among people with multiple sclerosis: a Swedish cohort study
Source: J Neurol Neurosurg Psychiatry. 2022 Dec 20;94(5):337–48. doi: 10.1136/jnnp-2022-329886 (PMC10176386; doi:10.1136/jnnp-2022-329886)
Supplement: Supplementary data [file jnnp-2022-329886supp003.pdf]

Supplementary Table 2: Association of relapses to baclofen treatment among incident and prevalent MS individuals.

			M1		M2		M3		M4		M5	
**Incident MS**	**E**	**PT**	**HR**	**95% CI**	**HR**	**95% CI**	**HR**	**95% CI**	**HR**	**95% CI**	**HR**	**95% CI**
**Relapses**												
No relapses	7	609	1.00		1.00		1.00		1.00		1.00	
1 relapse	56	4396	0.94	0.43,2.07	1.22	0.46,3.22	2.13	0.21,21.10	1.10	0.41,2.94	1.12	0.42,2.99
2 relapses	23	2054	0.98	0.41,2.31	1.35	0.49,3.74	2.52	0.25,24.87	1.09	0.38,3.10	1.10	0.39,3.15
**Age at MS onset**	82	6669			1.00	0.97,1.03	1.03	0.98,1.09	0.99	0.96,1.03	1.00	0.96,1.04
**EDSS at study entry**												
EDSS 0-2.5	22	2963					1.00					
EDSS 3-5.5	12	617					2.11	0.99,4.49				
EDSS 6+	1	35					1.98	0.21,18.59				
**DMT use**												
No DMT	3	659							1.00		1.00	
Moderately effective DMT	63	5275							2.70	0.83,8.79	2.77	0.82,9.33
Highly effective DMT	8	493							4.90	1.25,19.17	4.95	1.22,20.14

Individuals with incident MS diagnosed with RRMS with relapses (N=1146). Association of relapses at study entry to baclofen treatment. Model 1 adjusted for age, county of residence at MS diagnosis, and highest attained education. Model 2-5 additionally adjusted for number of years from MS onset to diagnosis and calendar year of MS diagnosis. Model 5 additionally adjusted for time with MS as an additional timescale. Disease modifying treatments are time-dependent variables. Note: varying number of individuals in each model due to missing values; Model 1 N=1146; Model 2 N=1077; Model 3 N=568; Model 4-5 N=908. Abbreviations: CI= confidence interval; DMT=disease modifying therapy; E=number of events; EDSS=Expanded Disability Severity Scale; HR=hazard ratio; M=model; PT=person-time. Reference category indicated by a hazard ratio of 1.00 with no confidence interval.

			M1		M2		M3		M4		M5	
**Prevalent MS**	**E**	**PT**	**HR**	**95% CI**	**HR**	**95% CI**	**HR**	**95% CI**	**HR**	**95% CI**	**HR**	**95% CI**
**Relapses**												
No relapse	11	516	1.00		1.00		1.00		1.00		1.00	
1 relapse	114	8053	0.75	0.40,1.41	0.77	0.41,1.44	1.25	0.30,5.23	0.88	0.44,1.75	0.90	0.45,1.79
2 relapses	33	2578	0.70	0.35,1.40	0.71	0.36,1.42	1.20	0.28,5.15	0.81	0.38,1.71	0.82	0.39,1.74
3 relapses	24	1552	0.83	0.40,1.72	0.85	0.41,1.76	1.03	0.23,4.63	0.88	0.40,1.93	0.89	0.40,1.96
4+ relapses	35	2445	0.79	0.40,1.58	0.80	0.40,1.58	1.44	0.34,6.13	0.90	0.43,1.91	0.92	0.43,1.94
**Age at MS diagnosis**	217	15144			0.99	0.95,1.04	0.99	0.94,1.05	0.99	0.95,1.04	1.05	0.94,1.18
**EDSS at study entry**												
EDSS 0-2.5	84	7629					1.00					
EDSS 3-5.5	43	2402					1.61	1.10,2.35				
EDSS 6+	11	155					6.54	3.44,12.45				
**DMT use**												
No DMT	16	1852							1.00		1.00	
Moderately effective DMT	183	11890							1.86	1.08,3.22	1.85	1.07,3.21
Highly effective DMT	5	240							2.81	1.00,7.88	2.77	0.99,7.79

Individuals with prevalent MS diagnosed with RRMS with relapses (N=1890). Association of relapses at study entry to baclofen treatment. Model 1 adjusted for age, county of residence at MS diagnosis, and highest attained education. Model 2-5 additionally adjusted for number of years from MS onset to diagnosis and calendar year of MS diagnosis. Model 5 additionally adjusted for time with MS as an additional timescale. Disease modifying treatments are time-dependent variables. Note: varying number of individuals in each model due to missing values. Model 1,2 N=1890, Model 3 N=1268 Models 4-5 N=1745. Abbreviations: CI= confidence interval; DMT=disease modifying therapy; E=number of events; EDSS=Expanded Disability Severity Scale; HR=hazard ratio; M=model; PT=person-time. Reference category indicated by a hazard ratio of 1.00 with no confidence interval.
