# Supplementary material for: Spasticity treatment patterns among people with multiple sclerosis: a Swedish cohort study
Source: J Neurol Neurosurg Psychiatry. 2022 Dec 20;94(5):337–48. doi: 10.1136/jnnp-2022-329886 (PMC10176386; doi:10.1136/jnnp-2022-329886)
Supplement: Supplementary data [file jnnp-2022-329886supp002.pdf]

Supplementary Table 1: Baseline characteristics assessed at study entry of incident and prevalent individuals with MS, stratified by use of treatments for spasticity.

	Incident		*Prevalent*	
Baseline characteristics	***No Spasticity***	***Spasticity***	***No Spasticity***	***Spasticity***
**N**	**1376**	**450**	**2347**	**1172**
**Type of treatment** n(%)				
Baclofen		138(30.67)		512(43.69)
Clonazepam		6(1.33)		39(3.33)
Diazepam		78(17.33)		231(19.71)
Gabapentin		226(50.22)		388(33.11)
Cannaboids		2(0.44)		2(0.17)
**Sex** n(%)				
Male	376(27.33)	133(29.56)	578(24.63)	324(27.65)
Female	1000(72.67)	317(70.44)	1769(75.37)	848(72.35)
**Highest attained education** n(%)				
Primary education	98(7.12)	55(12.22)	269(11.46)	193(16.47)
Secondary education	637(46.29)	227(50.44)	1087(46.31)	534(45.56)
Tertiary education	641(46.58)	168(37.33)	991(42.22)	445(37.97)
**Comorbid diseases** n(%)				
Amyotrophic lateral sclerosis	0(0.00)	0(0.00)	0(0.00)	1(0.09)
Depression	220(15.99)	114(25.33)	496(21.13)	384(32.76)
Concussion or TBI	2(0.15)	1(0.22)	4(0.17)	4(0.34)
Coronary artery disease	1(0.07)	1(0.22)	18(0.77)	10(0.85)
Vascular disease	13(0.94)	9(2.00)	68(2.90)	44(3.75)
Stroke	12(0.87)	4(0.89)	38(1.62)	21(1.79)
Parkinson's disease	3(0.22)	2(0.44)	4(0.17)	11(0.94)
Diabetes				
No diabetes	1364(99.13)	445(98.89)	2317(98.72)	1157(98.72)
Type 1	2(0.15)	4(0.89)	10(0.43)	6(0.51)
Type 2	9(0.65)	1(0.22)	18(0.77)	8(0.68)
Type unknown	1(0.07)	0(0.00)	2(0.09)	1(0.09)
Seizure	16(1.16)	6(1.33)	25(1.07)	24(2.05)
Epilepsy	9(0.65)	3(0.67)	18(0.77)	17(1.45)
**MS Characteristics** n(%)				
Year of MS diagnosis				
1945-1980	0(0.00)	0(0.00)	115(4.90)	47(4.01)
1981-1990	0(0.00)	0(0.00)	241(10.27)	121(10.32)
1991-2000	0(0.00)	0(0.00)	886(37.75)	501(42.75)
2001-2005	117(8.50)	38(8.44)	1105(47.08)	503(42.92)
2006-2007	433(31.47)	177(39.33)	0(0.00)	0(0.00)
2008-2009	357(25.94)	142(31.56)	0(0.00)	0(0.00)
2010-2011	329(23.91)	70(15.56)	0(0.00)	0(0.00)
2012-2014	140(10.17)	23(5.11)	0(0.00)	0(0.00)
MS Disease course n(%)				
Relapsing remitting	1154(83.87)	312(69.33)	1545(65.83)	577(49.23)
Primary progressive	52(3.78)	58(12.89)	138(5.88)	133(11.35)
Secondary progressive	67(4.87)	54(12.00)	589(25.10)	432(36.86)
Unknown	103(7.49)	26(5.78)	75(3.20)	30(2.56)
Mean age at MS diagnosis, years(SD, min-max)	38.93(0.29, 18.06-64.82)	42.44(0.49, 18.02-64.71)	38.93(0.22, 18.04-64.88)	40.47(0.31, 18.09-64.86)
Onset n (% missing)	1229(10.68)	407(9.56)	2115(9.88)	1069(8.79)
Mean age at MS onset, years(SD, min-max)	34.74(0.29, 8.75-63.59)	38.01(0.51, 10.37-62.67)	32.54(0.21, 6.13-62.92)	33.65(0.31, 11.75-62.46)
Number of relapses n (% missing)	1007(26.82)	323(28.22)	1941(17.30)	927(20.90)
0 relapses	104(10.33)	29(8.98)	62(3.19)	30(3.24)
1 relapse	624(61.97)	205(63.47)	1125(57.96)	574(61.92)

	Incident		*Prevalent*	
Baseline characteristics	***No Spasticity***	***Spasticity***	***No Spasticity***	***Spasticity***
2 relapses	190(18.87)	54(16.72)	294(15.15)	121(13.05)
3 relapses	55(5.46)	22(6.81)	182(9.38)	75(8.09)
4+ relapses	34(3.38)	13(4.02)	278(14.32)	127(13.70)
First DMT n (% missing)*	1239(9.96)	336(25.33)	2011(14.32)	917(21.76)
No DMT use	58(4.68)	29(8.63)	357(17.73)	158(17.21)
Moderately effective DMT	1032(83.29)	267(79.46)	1595(79.20)	740(80.61)
Highly effective DMT	149(12.03)	40(11.90)	62(3.08)	20(2.18)
Median years to first DMT, (IQR)	0.14(0.05-0.37)	0.11(0.05-0.29)	1.00(0.19-4.93)	1.05(0.14-4.75)
EDSS n (% missing)	618(55.09)	196(56.44)	1473(37.24)	714(39.08)
0	140(22.65)	15(7.65)	219(14.87)	42(5.88)
1-1.5	203(32.85)	43(21.94)	329(22.34)	90(12.61)
2-2.5	158(25.57)	67(34.18)	302(20.50)	129(18.07)
3-3.5	81(13.11)	40(20.41)	239(16.23)	111(15.55)
4-4.5	26(4.21)	13(6.63)	119(8.08)	68(9.52)
5-5.5	5(0.81)	7(3.57)	77(5.23)	48(6.72)
6-6.5	4(0.65)	10(5.10)	131(8.89)	169(23.67)
7-9.5	1(0.16)	1(0.51)	57(3.87)	57(7.98)

Abbreviations: %=percentage; +=or more; DMT=disease modifying therapy; EDSS=expanded disability severity scale HR=hazard ratio; IQR=interquartile range; max=maximum; min=minimum; N=total number individuals ; n=total number in subgroup; Ref=reference group; SD=standard deviation; TBI=traumatic brain injury.

Note: For many of the MS characteristics, there were missing values in each of the variables of interest. The percentage missing are listed in the table for each given variable.

† Univariate Cox proportional hazard models were used to calculate hazard ratios using age as the underlying timescale. The hazard ratios can be interpreted as incidence rate ratios.

***** Given the status of disease modifying therapy (DMT) use can occur sometime after MS diagnosis, first DMT use was used as a time varying covariate (the value of the variable was allowed to change over time).
