# Supplementary material for: Spasticity treatment patterns among people with multiple sclerosis: a Swedish cohort study
Source: J Neurol Neurosurg Psychiatry. 2022 Dec 20;94(5):337–48. doi: 10.1136/jnnp-2022-329886 (PMC10176386; doi:10.1136/jnnp-2022-329886)
Supplement: Supplementary data [file jnnp-2022-329886supp004.pdf]

Supplementary Table 3: Individuals with incident MS and general variable associations with spasticity treatment.

			M1		M2		M3		M4*		M5		M6	
	Events	PT	HR	95% CI	HR	95% CI	HR	95% CI	HR	95% CI	HR	95% CI	HR	95% CI
**Sex**														
Male	132	2518	1.00		1.00		1.00		1.00		1.00		1.00	
Female	316	6701	0.93	0.76,1.14	0.97	0.79,1.19	1.07	0.87,1.32	1.03	0.83,1.28	1.08	0.87,1.32	1.08	0.88,1.33
**Education**														
Compulsory	55	697			1.00		1.00		1.00		1.00		1.00	
Post-compulsory	226	4421			0.66	0.49,0.89	0.70	0.52,0.94	0.65	0.48,0.89	0.73	0.54,0.99	0.72	0.53,0.98
Tertiary	167	4101			0.52	0.38,0.71	0.56	0.41,0.76	0.57	0.41,0.79	0.59	0.43,0.81	0.57	0.42,0.79
**Disease course †**														
RRMS	298	7358					1.00		1.00		1.00		1.00	
PPMS	57	417					3.18	2.34,4.34	2.85	2.06,3.95	2.75	2.02,3.75	3.09	2.27,4.22
SPMS	67	750					2.22	1.67,2.95	2.70	2.00,3.63	2.21	1.66,2.94	2.26	1.70,3.01
Unknown	26	694					0.89	0.59,1.33	0.80	0.48,1.36	0.89	0.60,1.33	0.88	0.59,1.32
**Age at MS onset**	405	8325							1.04	1.02,1.06				
**Years with MS †**														
<0.5	108	768									1.00			
>=0.5 to <1	46	806									0.40	0.28,0.57		
>=1 to <2	82	1613									0.35	0.26,0.47		
>=2 to <3	62	1471									0.30	0.22,0.40		
>=3 to <6	112	3206									0.24	0.18,0.31		
>=6	38	1356									0.18	0.13,0.27		
**MS diagnosis year**														
2005	38	1130											1.00	
2006-2007	177	3921											1.30	0.92,1.85
2008-2009	141	2402											1.76	1.23,2.53
2010-2011	70	1420											1.51	1.01,2.25
2012-2014	22	346											1.99	1.17,3.38

Abbreviations: CI=confidence interval; HR=hazard ratio; M=model; RRMS=relapsing remitting MS; PT=person-time; PPMS=primary progressive MS; SPMS; secondary progressive MS.

Individuals with incident MS (N=1822) additionally excluding individuals with PPMS/SPMS diagnosed <= 25 year of age (n=4). All models are adjusted for age and county of residence at MS diagnosis. Variable categories with hazard ratios of 1.00 without confidence intervals are reference categories.

***** Due to unknown MS onset dates, model 4 includes 1632/1822 individuals.

† Time varying covariates.

Supplementary Table 4: Individuals with prevalent MS and their risk of spasticity treatment for general predictors.

			M1		M2		M3		M4		M5*		M6		M7	
	Events	PT	HR	95% CI	HR	95% CI	HR	95% CI	HR	95% CI	HR	95% CI	HR	95% CI	HR	95% CI
**Sex**																
Male	324	6015	1.00		1.00		1.00		1.00		1.00		1.00		1.00	
Female	844	17875	0.88	0.77,1.00	0.89	0.78,1.01	0.95	0.83,1.08	0.96	0.84,1.09	0.96	0.84,1.10	0.96	0.84,1.09	0.94	0.83,1.07
**Education**																
Compulsory	192	2908			1.00		1.00		1.00		1.00		1.00		1.00	
Post-compulsory	532	11036			0.75	0.63,0.89	0.78	0.66,0.92	0.78	0.66,0.92	0.74	0.62,0.88	0.78	0.66,0.92	0.77	0.65,0.92
Tertiary	444	9946			0.71	0.60,0.85	0.76	0.63,0.90	0.76	0.64,0.90	0.72	0.61,0.87	0.76	0.64,0.90	0.75	0.63,0.90
**Disease course** †																
RRMS	489	14255					1.00		1.00		1.00		1.00		1.00	
PPMS	130	1543					2.77	2.25,3.41	2.70	2.19,3.33	2.68	2.15,3.33	2.73	2.21,3.36	2.72	2.21,3.35
SPMS	519	7360					2.23	1.95,2.56	2.31	2.01,2.66	2.32	2.01,2.69	2.29	1.99,2.62	2.24	1.95,2.58
Unknown	30	732					1.28	0.88,1.85	1.23	0.84,1.78	0.93	0.52,1.66	1.24	0.85,1.79	1.26	0.87,1.83
**Age at MS diagnosis**	1168	23890							1.02	1.01,1.03						
**Age at MS onset**	1065	21573									1.01	1.00,1.02				
**Years with MS** †																
1 to <1.5	2	43											1.00			
>=1.5 to <2	9	119											1.57	0.34,7.32		
>=2 to <2.5	14	187											1.54	0.35,6.82		
>=2 to <3	15	256											1.19	0.27,5.20		
>=3 to <3.5	18	316											1.13	0.26,4.88		
>=3.5 to 4	20	388											0.98	0.23,4.21		
>=4 to <6	113	2414											0.89	0.22,3.61		
>=6 to <8	168	3339											0.92	0.23,3.74		
>=8 to <10	173	3759											0.83	0.20,3.35		
>=10	636	13069											0.79	0.20,3.18		
**MS diagnosis year**																
1945-1980	47	1092													0.81	0.59,1.12
1981-1990	121	2409													0.89	0.72,1.10
1991-2000	501	9209													1.08	0.95,1.23
2001-2005	499	11179													1.00	

Abbreviations: CI=confidence interval; HR=hazard ratio; M=model; RRMS=relapsing remitting MS; PT=person-time; PPMS=primary progressive MS; SPMS; secondary progressive MS.

Individuals with prevalent MS (N=3514) additionally excluding individuals with PPMS/SPMS under age 25 at study entry (n=5). All models adjusted for age and county of residence at MS diagnosis. Disease course and years with MS are time-varying covariates. Reference categories indicated by hazard ratios of 1.00 with no confidence interval.

***** Due to unknown MS onset dates, model 5 includes 3179/3514 individuals.

† Time varying covariates.

Smith KA, *et al. J Neurol Neurosurg Psychiatry* 2023; 94:1–12. doi: 10.1136/jnnp-2022-329886

Supplementary Table 5: Sensitivity analysis among incident MS individuals with no diagnosis of depression or prescription for antidepressants. General variables and their association with spasticity treatment.

			M1		M2		M3		M4 *		M5		M6	
	Events	PT	HR	95% CI	HR	95% CI	HR	95% CI	HR	95% CI	HR	95% CI	HR	95% CI
**Sex**														
Male	78	1725	1.00		1.00		1.00		1.00		1.00		1.00	
Female	169	3815	1.00	0.76,1.32	1.06	0.80,1.39	1.17	0.88,1.55	1.14	0.84,1.54	1.22	0.92,1.63	1.21	0.91,1.61
**Education**														
Compulsory	27	334			1.00		1.00		1.00		1.00		1.00	
Post-compulsory	120	2612			0.59	0.38,0.92	0.71	0.45,1.11	0.68	0.42,1.09	0.73	0.46,1.16	0.71	0.45,1.13
Tertiary	100	2594			0.49	0.31,0.77	0.57	0.36,0.91	0.61	0.38,1.00	0.60	0.38,0.97	0.57	0.36,0.90
**Disease course** †														
RRMS	165	4637					1.00		1.00		1.00		1.00	
PPMS	33	200					5.06	3.27,7.82	4.73	3.01,7.43	3.92	2.51,6.12	4.84	3.13,7.48
SPMS	37	363					2.86	1.92,4.25	3.66	2.39,5.59	2.85	1.90,4.27	2.75	1.84,4.10
Unknown	12	340					1.08	0.59,1.96	0.83	0.36,1.89	1.05	0.58,1.93	1.07	0.59,1.95
**Age at MS onset**	223	5073							1.06	1.03,1.09				
**Years with MS** †														
<0.5	76	475									1.00			
>=0.5 to <1	29	494									0.42	0.27,0.65		
>=1 to <2	43	983									0.28	0.19,0.42		
>=2 to <3	32	893									0.23	0.15,0.35		
>=3 to <6	54	1912									0.17	0.12,0.25		
>=6	13	782									0.09	0.05,0.18		
**MS diagnosis year**														
2005	18	624											1.00	
2006-2007	95	2229											1.41	0.84,2.37
2008-2009	81	1521											1.87	1.10,3.18
2010-2011	40	920											1.54	0.87,2.73
2012-2014	13	244											1.94	0.93,4.05

Abbreviations: CI=confidence interval; HR=hazard ratio; M=model; RRMS=relapsing remitting MS; PT=person-time; PPMS=primary progressive MS; SPMS; secondary progressive MS.

Individuals with incident MS (N=1123) excluding individuals (n=703) ever diagnosed with depression or prescribed an anti-depressant and PPMS/SPMS diagnosed < age 25 year (n=2). All models adjusted for age and county of residence at MS diagnosis. In models where region of residence violated proportional hazards assumption, a stratified Cox model was used. Variable categories with hazard ratios of 1.00 without confidence intervals are reference categories.

* Due to missing data, model including MS onset includes N=1011.

† Time varying covariates.

Supplementary Table 6: Sensitivity analysis among incident MS individuals excluding individuals with a history of seizures for general variable asssociations with spasticity treatment.

			M1		M2		M3		M4*		M5		M6	
	Events	PT	HR	95% CI	HR	95% CI	HR	95% CI	HR	95% CI	HR	95% CI	HR	95% CI
**Sex**														
Male	131	2462	1.00		1.00		1.00		1.00		1.00		1.00	
Female	308	6586	0.91	0.74,1.12	0.95	0.77,1.18	1.04	0.84,1.28	0.98	0.79,1.22	1.06	0.86,1.31	1.05	0.85,1.30
**Education**														
Compulsory	55	673			1.00		1.00		1.00		1.00		1.00	
Post-compulsory	219	4323			0.62	0.45,0.84	0.64	0.47,0.87	0.61	0.45,0.85	0.68	0.50,0.93	0.66	0.49,0.90
Tertiary	165	4051			0.50	0.36,0.69	0.53	0.38,0.73	0.56	0.40,0.78	0.56	0.41,0.77	0.54	0.39,0.75
**Disease course** †														
RRMS	291	7231					1.00		1.00		1.00		1.00	
PPMS	56	413					3.24	2.36,4.45	2.98	2.13,4.16	2.71	1.97,3.74	3.13	2.28,4.30
SPMS	66	729					2.30	1.72,3.07	2.71	2.00,3.68	2.28	1.70,3.05	2.36	1.76,3.15
Unknown	26	674					0.97	0.64,1.45	0.90	0.53,1.52	0.96	0.64,1.44	0.96	0.64,1.44
**Age at MS onset**	397	8178							1.04	1.02,1.06				
**Years with MS** †														
<0.5	105	754									1.00			
>=0.5 to <1	46	792									0.46	0.32,0.65		
>=1 to <2	82	1585									0.40	0.30,0.54		
>=2 to <3	60	1444									0.31	0.22,0.42		
>=3 to <6	109	3140									0.25	0.19,0.33		
>=6	37	1333									0.19	0.13,0.28		
**MS diagnosis year**														
2005	37	1118											1.00	
2006-2007	172	3824											1.30	0.91,1.86
2008-2009	138	2355											1.72	1.19,2.49
2010-2011	70	1405											1.52	1.02,2.29
2012-2014	22	346											2.20	1.29,3.77

Abbreviations: CI=confidence interval; HR=hazard ratio; M=model; RRMS=relapsing remitting MS; PT=person-time; PPMS=primary progressive MS; SPMS; secondary progressive MS.
Individuals with incident MS (N=1790) excluding individuals ever diagnosed with seizures (n=32) and PPMS/SPMS individuals <= 25 years of age at MS diagnosis (n=4).
Adjusted for age and county of residence at MS diagnosis. In models where region of residence violated the proportional hazards assumption, stratified Cox regression models were used. Variable categories with hazard ratios of 1.00 without confidence intervals are reference categories.
*Model with MS onset includes N=1605 individuals due to missing onset dates.
† Time-varying covariates.

Supplementary Table 7: Sensitivity analysis among individuals with prevalent MS with no diagnosis of depression or prescription for antidepressants. General variables and their association with spasticity treatment.

			M1		M2		M3		M4		M5*		M6		M7	
	**Events**	**PT**	**HR**	**95% CI**	**HR**	**95% CI**	**HR**	**95% CI**	**HR**	**95% CI**	**HR**	**95% CI**	**HR**	**95% CI**	**HR**	**95% CI**
**Sex**																
Male	199	4045	1.00		1.00		1.00		1.00		1.00		1.00		1.00	
Female	412	10030	0.82	0.69,0.98	0.83	0.70,0.99	0.91	0.77,1.09	0.92	0.78,1.10	0.92	0.77,1.10	0.93	0.78,1.11	0.92	0.77,1.09
**Education**																
Compulsory	94	1684			1.00		1.00		1.00		1.00		1.00		1.00	
Post-compulsory	279	6382			0.78	0.61,0.99	0.85	0.67,1.08	0.85	0.67,1.08	0.81	0.63,1.05	0.85	0.67,1.09	0.84	0.66,1.07
Tertiary	238	6009			0.72	0.56,0.93	0.78	0.61,1.01	0.78	0.61,1.00	0.76	0.59,0.99	0.79	0.61,1.01	0.78	0.61,1.00
**Disease course** [†]																
RRMS	239	8575					1.00		1.00		1.00		1.00		1.00	
PPMS	80	1029					3.27	2.47,4.33	3.20	2.42,4.23	3.20	2.39,4.27	3.22	2.44,4.27	3.25	2.45,4.30
SPMS	273	4034					2.76	2.27,3.36	2.91	2.39,3.55	2.93	2.38,3.62	2.92	2.39,3.56	2.79	2.29,3.40
Unknown	19	437					1.78	1.11,2.86	1.69	1.05,2.73	1.50	0.78,2.86	1.68	1.04,2.71	1.76	1.09,2.83
**Age at MS diagnosis**	611	14075							1.02	1.01,1.03						
**Age at MS onset**	559	12768									1.01	1.00,1.02				
**Years with MS**[†]																
1 to <1.5	1	26											1.00			
>=1.5 to <2	5	74											1.77	0.20,15.63		
>=2 to <2.5	9	118											1.98	0.24,16.05		
>=2 to <3	12	159											2.11	0.27,16.65		
>=3 to <3.5	10	194											1.30	0.16,10.46		
>=3.5 to 4	12	237											1.12	0.14,8.82		
>=4 to <6	64	1428											1.05	0.14,7.80		
>=6 to <8	99	1960											1.14	0.15,8.37		
>=8 to <10	84	2232											0.80	0.11,5.88		
>=10	315	7646											0.78	0.11,5.74		
**MS diagnosis year**																
1945-1980	24	673													0.81	0.51,1.26
1981-1990	68	1425													0.92	0.70,1.23
1991-2000	252	5377													1.01	0.84,1.20
2001-2005	267	6599													1.00	

Abbreviations: CI=confidence interval; HR=hazard ratio; M=model; RRMS=relapsing remitting MS; PT=person-time; PPMS=primary progressive MS; SPMS; secondary progressive MS.

Individuals with prevalent MS (N=2040) excluding individuals ever diagnosed with depression or prescribed an antidepressant (n=1479) and PPMS/SPMS individuals diagnosed < age 25 years (n=5). All models adjusted for age and county of residence at MS diagnosis. In models where region of residence violated the proportional hazards assumption, a stratified Cox model was used. Categories with HR of 1.00 and no confidence intervals are reference categories.

***** Due to unknown MS onset dates, model 5 includes 1851/2040 individuals.

[†] Time varying covariates.

Supplementary Table 8: Sensitivity analysis among individuals with prevalent MS with no diagnosis of seizures. General variables and their association with spasticity treatment.

			**M1**		**M2**		**M3**		**M4**		**M5***		**M6**		**M7**	
	**Events**	**PT**	**HR**	**95% CI**	**HR**	**95% CI**	**HR**	**95% CI**	**HR**	**95% CI**	**HR**	**95% CI**	**HR**	**95% CI**	**HR**	**95% CI**
**Sex**																
Male	317	5923	1.00		1.00		1.00		1.00		1.00		1.00		1.00	
Female	814	17475	0.87	0.77,0.99	0.89	0.78,1.01	0.94	0.82,1.07	0.95	0.83,1.08	0.96	0.84,1.10	0.95	0.83,1.08	0.94	0.82,1.07
**Education**																
Compulsory	187	2831			1.00		1.00		1.00		1.00		1.00		1.00	
Post-compulsory	517	10821			0.74	0.63,0.88	0.77	0.65,0.91	0.77	0.65,0.91	0.73	0.61,0.87	0.77	0.65,0.92	0.76	0.64,0.91
Tertiary	427	9746			0.70	0.59,0.84	0.74	0.62,0.89	0.75	0.62,0.89	0.72	0.60,0.86	0.75	0.63,0.89	0.74	0.62,0.89
**Disease course** [†]																
RRMS	480	14031					1.00		1.00		1.00		1.00		1.00	
PPMS	121	1497					2.64	2.13,3.27	2.57	2.07,3.19	2.56	2.05,3.20	2.60	2.10,3.22	2.60	2.09,3.22
SPMS	503	7158					2.22	1.93,2.55	2.31	2.01,2.65	2.29	1.97,2.66	2.27	1.98,2.62	2.24	1.94,2.57
Unknown	27	712					1.19	0.80,1.75	1.14	0.77,1.68	0.95	0.53,1.70	1.15	0.78,1.70	1.17	0.79,1.73
**Age at MS diagnosis**	1131	23398							1.02	1.01,1.03						
**Age at MS onset**	1034	21142									1.01	1.00,1.02				
**Years with MS** [†]																
1 to <1.5	2	42											1.00			
>=1.5 to <2	8	117											1.40	0.30,6.65		
>=2 to <2.5	13	184											1.42	0.32,6.32		
>=2 to <3	15	251											1.18	0.27,5.17		
>=3 to <3.5	18	310											1.13	0.26,4.89		
>=3.5 to 4	20	379											0.98	0.23,4.22		
>=4 to <6	110	2361											0.87	0.21,3.52		
>=6 to <8	160	3269											0.88	0.22,3.56		
>=8 to <10	171	3691											0.81	0.20,3.28		
>=10	614	12794											0.76	0.19,3.04		
**Year of MS diagnosis**																
1945-1980	46	1053													0.81	0.59,1.12
1981-1990	116	2348													0.88	0.71,1.08
1991-2000	484	9050													1.07	0.94,1.21
2001-2005	485	10947													1.00	

Abbreviations: CI=confidence interval; HR=hazard ratio; M=model; RRMS=relapsing remitting MS; PT=person-time; PPMS=primary progressive MS; SPMS; secondary progressive MS.

Individuals with prevalent MS (N=3434) excluding individuals ever diagnosed with seizure (n=80) and PPMS/SPMS individuals <= 25 years of age at study entry (n=5). Adjusted for age and county of residence at MS diagnosis. Disease course and years with MS are time-varying covariates. Reference categories indicated by hazard ratios of 1.00 with no confidence interval.

***** Due to unknown MS onset dates, model 5 includes 3109/3434 individuals.

[†] Time varying covariates.

Supplementary Table 9: Expanded disability severity scores as fixed and time-varying covariates, disease course and disease modifying therapies and their association with spasticity treatment among individuals with incident MS.

**Baseline EDSS scores**														
			**M1**		**M2**		**M3**		**M4**		**M5**		**M6**	
**EDSS**	**Events**	**PT**	**HR**	**95% CI**	**HR**	**95% CI**	**HR**	**95% CI**	**HR**	**95% CI**	**HR**	**95% CI**	**HR**	**95% CI**
0	15	903	1.00		1.00		1.00		1.00		1.00		1.00	
1-1.5	43	1324	1.78	0.98,3.23	1.70	0.92,3.14	1.62	0.87,3.00	1.61	0.87,2.99	1.47	0.76,2.83	1.47	0.76,2.82
2-2.5	66	1107	3.23	1.83,5.71	3.55	1.97,6.39	3.51	1.95,6.31	3.37	1.88,6.07	2.84	1.52,5.30	2.76	1.48,5.16
3-3.5	39	571	3.63	1.97,6.68	3.64	1.93,6.84	3.48	1.85,6.56	3.27	1.73,6.17	2.67	1.33,5.37	2.63	1.31,5.29
4+	31	266	6.18	3.25,11.75	5.39	2.75,10.56	5.68	2.90,11.15	4.54	2.29,9.03	3.98	1.83,8.64	3.82	1.75,8.31
**Age at onset**	405	8325			1.05	1.02,1.07	1.05	1.02,1.08	1.07	1.04,1.10	1.03	1.00,1.06	1.03	1.00,1.06
**Disease course**														
RRMS	312	7540							1.00		1.00		1.00	
PPMS	57	417							1.71	0.97,3.02	1.07	0.44,2.56	1.11	0.46,2.65
SPMS	53	569							2.88	1.77,4.66	2.26	1.26,4.05	2.15	1.19,3.87
Unknown	26	694							0.46	0.17,1.27	0.61	0.19,1.96	0.61	0.19,1.96
**DMT†**														
None	29	1144									1.00		1.00	
Mod effective	266	6443									1.59	0.88,2.86	1.78	0.97,3.25
High effective	40	643									1.90	0.94,3.84	2.18	1.06,4.50
**Time varying EDSS scores**														
			**TVC1**		**TVC2**		**TVC3**		**TVC4**		**TVC5**		**TVC6**	
**EDSS†**	**Events**	**PT**	**HR**	**95% CI**	**HR**	**95% CI**	**HR**	**95% CI**	**HR**	**95% CI**	**HR**	**95% CI**	**HR**	**95% CI**
No score	16	1076	1.00		1.00		1.00		1.00		1.00		1.00	
0	27	1605	1.18	0.64,2.20	1.35	0.69,2.63	1.37	0.70,2.68	1.44	0.74,2.80	2.64	1.13,6.17	3.24	1.37,7.62
1.0-1.5	56	2161	1.78	1.01,3.12	1.85	1.01,3.38	1.85	1.01,3.39	1.93	1.05,3.53	3.20	1.43,7.17	3.95	1.77,8.81
2.0-2.5	81	1641	3.23	1.87,5.58	3.47	1.92,6.26	3.45	1.91,6.22	3.48	1.93,6.26	5.69	2.59,12.49	7.03	3.19,15.50
3.0-3.5	57	922	4.04	2.27,7.19	4.35	2.33,8.14	4.28	2.28,8.02	4.27	2.28,7.99	7.65	3.38,17.29	9.25	4.11,20.82
4.0-4.5	27	306	5.92	3.13,11.20	7.03	3.61,13.69	6.99	3.58,13.64	6.44	3.28,12.66	10.92	4.46,26.77	13.30	5.46,32.38
5.0-5.5	13	123	6.80	3.16,14.61	8.11	3.33,19.71	7.93	3.21,19.60	6.92	2.78,17.25	11.79	4.02,34.61	15.96	5.70,44.66
6.0-8.5	45	259	11.31	6.15,20.79	11.20	5.72,21.94	11.00	5.61,21.59	9.84	5.00,19.35	14.14	5.44,36.76	18.63	7.23,48.00
**Age at onset**	302	7528			1.04	1.02,1.07	1.04	1.02,1.07	1.05	1.03,1.07	1.04	1.02,1.07	1.02	1.00,1.05
**Disease course**														
RRMS	235	6802							1.00		1.00		1.00	
PPMS	38	358							1.45	0.90,2.33	1.10	0.56,2.15	1.19	0.62,2.25
SPMS	40	541							1.81	1.22,2.70	1.46	0.93,2.29	1.35	0.87,2.11
Unknown	9	391							0.59	0.27,1.27	0.59	0.25,1.44	0.59	0.24,1.45
**DMT†**														
None	27	1055									1.00		1.00	
Mod effective	199	5796									1.22	0.76,1.97	1.47	0.92,2.35
High effective	34	599									1.55	0.85,2.85	1.91	1.04,3.51

Abbreviations: CI=confidence interval; High=highly; HR=hazard ratio; M=model; Mod=moderately; RRMS=relapsing remitting MS; PT=person-time; PPMS=primary progressive MS; SPMS; secondary progressive MS; TVC=time-varying covariate.

Individuals with incident MS. Model 1 adjusted for age, county of residence at MS diagnosis, and highest attained education. Model 2 additionally adjusted for number of years from MS onset to diagnosis and calendar year of MS diagnosis. Model 3 additionally adjusted for vascular disease and depression. Model 6 additionally adjusted for time with MS as an additional timescale. Note, due to missing values, the number of individuals in each model vary. Baseline EDSS: Model 1 N=811, Models 2-4 N=764, Models 5-6 N=670. Time-varying EDSS: TVC 1 N=1510, TVC 2-4 N=1398, and TVC 5-6 N=1258. More individuals were included in the TVC EDSS models as not all had baseline EDSS scores, but were assessed for their EDSS scores after baseline. † Time-varying covariates.
