# Supplementary material for: Spasticity treatment patterns among people with multiple sclerosis: a Swedish cohort study
Source: J Neurol Neurosurg Psychiatry. 2022 Dec 20;94(5):337–48. doi: 10.1136/jnnp-2022-329886 (PMC10176386; doi:10.1136/jnnp-2022-329886)
Supplement: Supplementary data [file jnnp-2022-329886supp005.pdf]

Supplementary Table 10: Comorbid diseases as risk factors for spasticity treatment among individuals with incident and prevalent MS.

			M1		M2		M3	
Incident MS (N=1822)	**Events**	**PT**	**HR**	**95% CI**	**HR**	**95% CI**	**HR**	**95% CI**
**Concussion or TBI***			/	/	/	/	/	/
**Coronary artery disease**	3	25	2.04	0.83,4.98	2.17	0.86,5.43	2.83	1.12,7.18
**Depression**	198	2878	1.66	1.37,2.01	1.59	1.31,1.93	1.83	1.50,2.23
**Diabetes**								
Type I	5	17	3.06	1.32,7.11	2.73	1.17,6.34	2.68	1.12,6.39
Type II/Unknown	2	59	0.58	0.18,1.83	0.53	0.16,1.69	0.60	0.19,1.93
**Parkinson's disease**	2	10	3.20	1.47,6.94	3.61	1.65,7.89	3.55	1.62,7.75
**Seizure**	9	147	1.47	0.75,2.87	1.52	0.78,2.98	1.68	0.86,3.30
**Stroke**	3	83	0.80	0.35,1.81	0.88	0.38,2.01	0.91	0.40,2.10
**Vascular disease**	16	123	2.02	1.31,3.12	2.09	1.34,3.27	2.32	1.48,3.64
Prevalent MS (N=3514)								
**Concussion or TBI**	5	54	1.73	0.86,3.51	1.47	0.73,2.98	1.48	0.73,2.99
**Coronary artery disease**	15	239	1.20	0.80,1.79	1.20	0.80,1.80	1.22	0.81,1.83
**Depression**	544	7885	1.76	1.56,1.97	1.72	1.53,1.94	1.74	1.55,1.95
**Diabetes**								
Type I	2	56	1.14	0.54,2.41	1.25	0.59,2.64	1.26	0.60,2.65
Type II / Unknown	8	164	0.87	0.53,1.44	0.83	0.50,1.36	0.83	0.51,1.37
**Parkinson's disease**	8	94	1.46	0.95,2.26	1.41	0.91,2.18	1.41	0.91,2.18
**Seizure**	37	369	2.06	1.48,2.87	1.87	1.35,2.61	1.89	1.36,2.63
**Stroke**	19	351	1.30	0.93,1.80	1.34	0.96,1.87	1.34	0.96,1.86
**Vascular disease**	42	845	1.23	0.99,1.54	1.20	0.96,1.50	1.21	0.97,1.51

Abbreviations: CI=confidence interval; HR=hazard ratio; M=model; PT=person-time; TBI=traumatic brain injury.

All risk factors for spasticity were used as time-varying covariates and models included each risk factor individually. Reference categories are individuals without the comorbid disease.

Model 1 adjusted for age, county of residence at MS diagnosis, and highest attained education. Model 2 additionally adjusted for disease course as a time-varying covariate. Model 3 additionally adjusted for years with MS as an additional timescale. Stratified Cox regression models were used in the incident MS cohort where region of residence did not meet the proportional hazards assumption and among the prevalent MS cohort where education did not meet proportional hazards assumption. Additional incident MS individuals (n=4) at MS diagnosis and prevalent MS individuals (n=5) at study entry with PPMS/SPMS <= 25 years of age were excluded.

*Concussion or TBI estimates could not be computed as few individuals experienced concussion or TBI.
