# Supplementary figures and images for: Spasticity treatment patterns among people with multiple sclerosis: a Swedish cohort study
Source: J Neurol Neurosurg Psychiatry. 2022 Dec 20;94(5):337–48. doi: 10.1136/jnnp-2022-329886 (PMC10176386; doi:10.1136/jnnp-2022-329886)

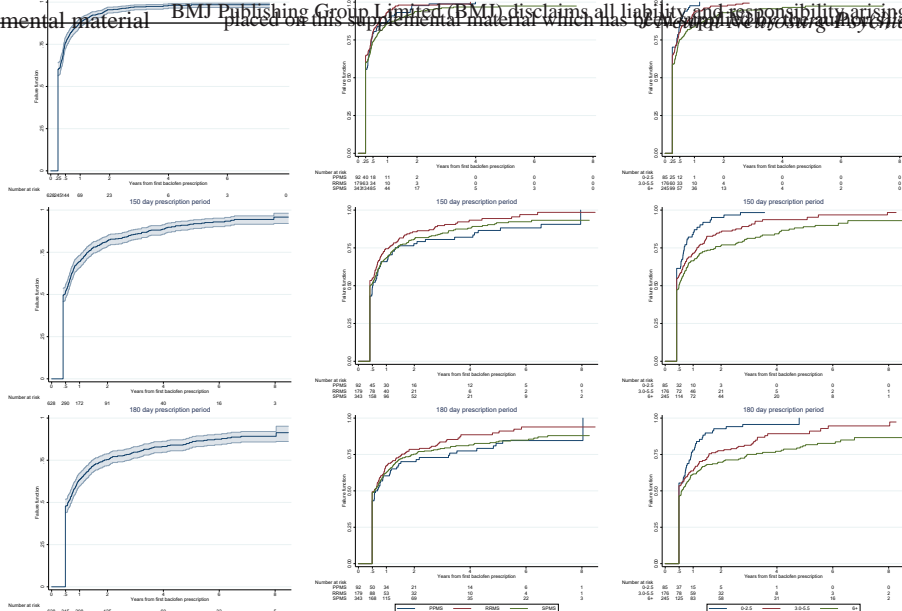

Supplement: Supplementary data [file jnnp-2022-329886supp006.pdf]
