# Supplementary material for: Spasticity treatment patterns among people with multiple sclerosis: a Swedish cohort study
Source: J Neurol Neurosurg Psychiatry. 2022 Dec 20;94(5):337–48. doi: 10.1136/jnnp-2022-329886 (PMC10176386; doi:10.1136/jnnp-2022-329886)
Supplement: Supplementary data [file jnnp-2022-329886supp007.pdf]

BMJ Publishing Group Limited (BMJ) disclaims all liability and responsibility arising from a
placed on this supplemental material which has been

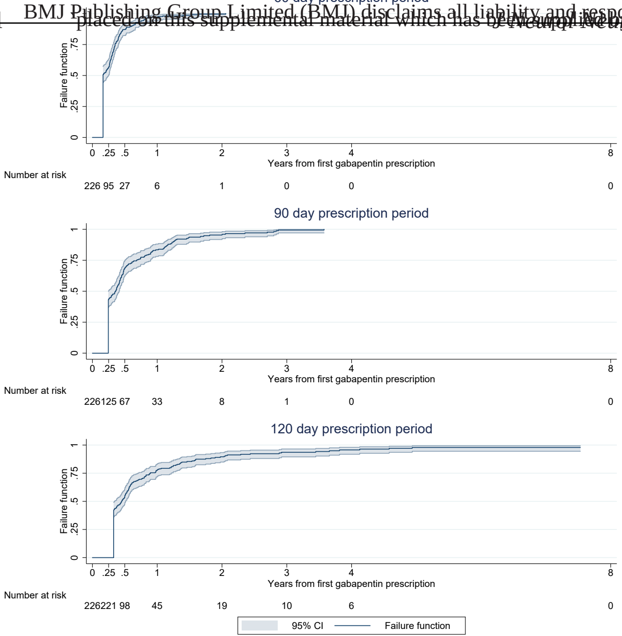

Supplementary Figure 4: Time to discontinuation of gabapentin among people with incident multiple sclerosis who had gabapentin as their first drug to treat possible spasticity.
