# Supplementary material for: Spasticity treatment patterns among people with multiple sclerosis: a Swedish cohort study
Source: J Neurol Neurosurg Psychiatry. 2022 Dec 20;94(5):337–48. doi: 10.1136/jnnp-2022-329886 (PMC10176386; doi:10.1136/jnnp-2022-329886)
Supplement: Supplementary data [file jnnp-2022-329886supp009.pdf]

BMJ Publishing Group Limited (BMJ) disclaims all liability and responsibility arising from a
placed on this supplemental material which has been

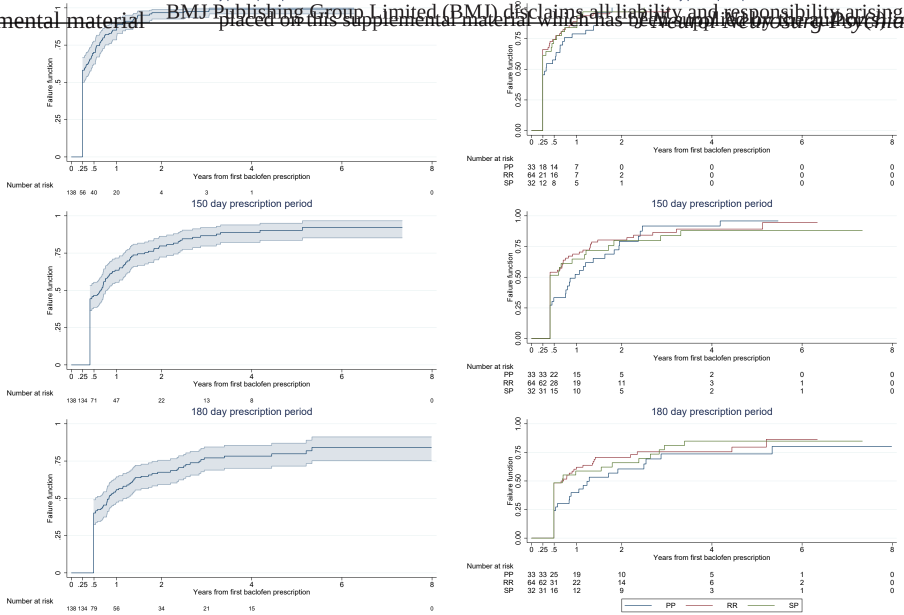

Supplementary Figure 4: Time to discontinuation of baclofen of people who first had baclofen prior to any other spasticity treatment among people with incident multiple sclerosis stratified by disease course.
Left column, overall discontinuation. Right column, discontinuation stratified by EDSS score when starting baclofen.
Abbreviations: RR = Relapsing-remitting multiple sclerosis; SP = secondary progressive multiple sclerosis; PP = primary progressive multiple sclerosis.
